# FR171456 is a specific inhibitor of mammalian NSDHL and yeast Erg26p

**DOI:** 10.1038/ncomms9613

**Published:** 2015-10-12

**Authors:** Stephen B. Helliwell, Shantanu Karkare, Marc Bergdoll, Alain Rahier, Juliet R. Leighton-Davis, Celine Fioretto, Thomas Aust, Ireos Filipuzzi, Mathias Frederiksen, John Gounarides, Dominic Hoepfner, Andreas Hofmann, Pierre-Eloi Imbert, Rolf Jeker, Richard Knochenmuss, Philipp Krastel, Anais Margerit, Klaus Memmert, Charlotte V. Miault, N. Rao Movva, Alban Muller, Hans-Ulrich Naegeli, Lukas Oberer, Vivian Prindle, Ralph Riedl, Sven Schuierer, Jessica A. Sexton, Jianshi Tao, Trixie Wagner, Hong Yin, Juan Zhang, Silvio Roggo, Stefan Reinker, Christian N. Parker

**Affiliations:** 1 Novartis Institutes for BioMedical Research, Novartis Campus, Basel, CH-4056, Switzerland; 2 Institut de Biologie Moléculaire des Plantes, CNRS, Unité Propre de Recherche 2357, Strasbourg cedex 67083, France; 3 Novartis Institutes for BioMedical Research, 250 Massachusetts Avenue, Cambridge, Massachusetts, 02139, USA; 4 GNF, San Diego, California 92121, USA

## Abstract

FR171456 is a natural product with cholesterol-lowering properties in animal models, but its molecular target is unknown, which hinders further drug development. Here we show that FR171456 specifically targets the sterol-4-alpha-carboxylate-3-dehydrogenase (S*accharomyces cerevisiae*—Erg26p, *Homo sapiens*—NSDHL (NAD(P) dependent steroid dehydrogenase-like)), an essential enzyme in the ergosterol/cholesterol biosynthesis pathway. FR171456 significantly alters the levels of cholesterol pathway intermediates in human and yeast cells. Genome-wide yeast haploinsufficiency profiling experiments highlight the *erg26/ERG26* strain, and multiple mutations in *ERG26* confer resistance to FR171456 in growth and enzyme assays. Some of these *ERG26* mutations likely alter Erg26 binding to FR171456, based on a model of Erg26. Finally, we show that FR171456 inhibits an artificial Hepatitis C viral replicon, and has broad antifungal activity, suggesting potential additional utility as an anti-infective. The discovery of the target and binding site of FR171456 within the target will aid further development of this compound.

## Abstract (web summary)

FR171456 is a bioactive chemical produced by some microorganisms. Here, the authors identify the enzyme NSDHL of the sterol synthesis pathway as the molecular target of FR171456, rendering it the first compound to specifically target this class of enzyme in yeast and mammalian cells.

## Graphical abstract

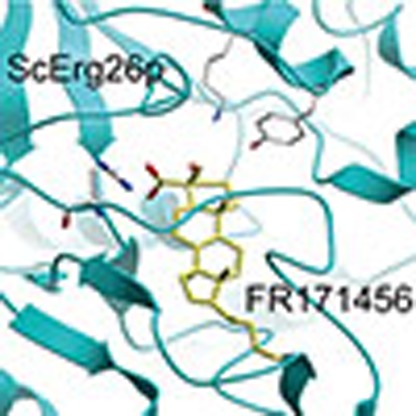

The cholesterol biosynthesis pathway, also known as the mevalonate pathway, is an essential cellular pathway that generates cholesterol—a major component of the plasma membrane. It represents a complex but highly regulated pathway, with the enzyme 3-hydroxy-3-methylglutaryl-coenzyme A reductase (HMGCR) being the most tightly regulated component controlling entry into the cholesterol pathway^1^. Cholesterol plays an important role in maintaining the plasma membrane integrity and dysregulation of this pathway has been shown to be a major cause of cardiovascular disease, thus contributing to mortality and morbidity worldwide^2^.

To counter cholesterol pathway imbalances in human disease, many molecules have been developed that target sterol biosynthesis enzymes. Statins target HMGCR, bisphosphonates target farnesyl diphosphate synthase, zaragozic acid and quinuclidines (3-(biphenyl-4-yl)-3-hydroxyquinuclidine) target squalene synthase. Further down the pathway, allylamines target squalene epoxidase, azoles target lanosterol 14α-demethylase, morpholines target sterol C_8_–C_7_ isomerase/sterol reductase and azasterol targets sterol 24-C-methyltransferase^3^,^4^,^5^,^6^,^7^. Statins have been extremely successful in treating hypercholesterolemia but a significant clinical population experience side effects that prevent continuous or further use^8^.

Most other sterol pathway inhibitors have proven to be unsuitable for widespread clinical application due to detrimental physiological side effects. A number of these agents target fungal-specific stages of the pathway and have found application as anti-fungals. However, their poor anti-fungal spectrum, and the development of resistance to these anti-fungal treatments limits their usefulness. There is thus a clinical need for inhibitors of other components of the cholesterol pathway.

Sterol-4-α-carboxylate 3-dehydrogenase, decarboxylating (NSDHL; often referred to as 3β-hydroxysteroid dehydrogenase/C4 decarboxylase [3βHSD/D]), is conserved amongst eukaryotes and lies in the cholesterol pathway. NSDHL is distal to lanosterol synthase, and catalyses NAD^+^-dependent oxidative decarboxylation of 4α–carboxysterol intermediates involved in the C-4 demethylation process of sterol precursors to produce the corresponding 3-keto, C-4-decarboxylated products^9^,^10^. The NSDHL equivalent in *S. cerevisiae*—Erg26p, encoded by the essential gene *ERG26*, also acts in the lanosterol–distal section of the yeast ergosterol synthesis pathway^11^. 3βHSD/D from *Arabidopsis thaliana* has been characterized and its enzymology has been studied^12^, and essential catalytic and binding residues have been identified^12^,^13^. According to a homology model and biochemical studies of the *A. thaliana* enzyme^13^, Tyr159 and Lys163 are oriented near the 3β-hydroxyl group of the substrate and directly involved in the dehydrogenation process, while Arg326 forms an essential salt bridge with the 4α-carboxyl group of the substrate. The Asp39 residue is thought to contact the hydroxyl groups of the adenosine-ribose ring of NAD^+^. These crucial residues are highly conserved across plant, fungal and mammalian enzymes.

This report describes the identification of the previously described natural product FR171456 (refs 14, 15) using bioactivity guided fractionation directed by inhibition of a Hepatitis C viral (HCV) replicon assay^16^. We used a range of complementary methods in three organisms to demonstrate that FR171456 targets the Erg26p/NSDHL enzyme of the sterol biosynthesis pathway. This is the first compound known to inhibit this enzyme specifically, and therefore represents a useful tool for chemical biologists. Since FR171456 targets a previously untargeted node in the sterol pathway these results may spur the development of a novel class of compounds with utility in hypercholesterolemia or fungal infection^17^. This discovery is entirely consistent with the compound's effects on cholesterol in rats and rabbits^14^,^15^.

## Results

### Metabolomics suggests that FR171456 inhibits NSDHL

**Figure 1:**
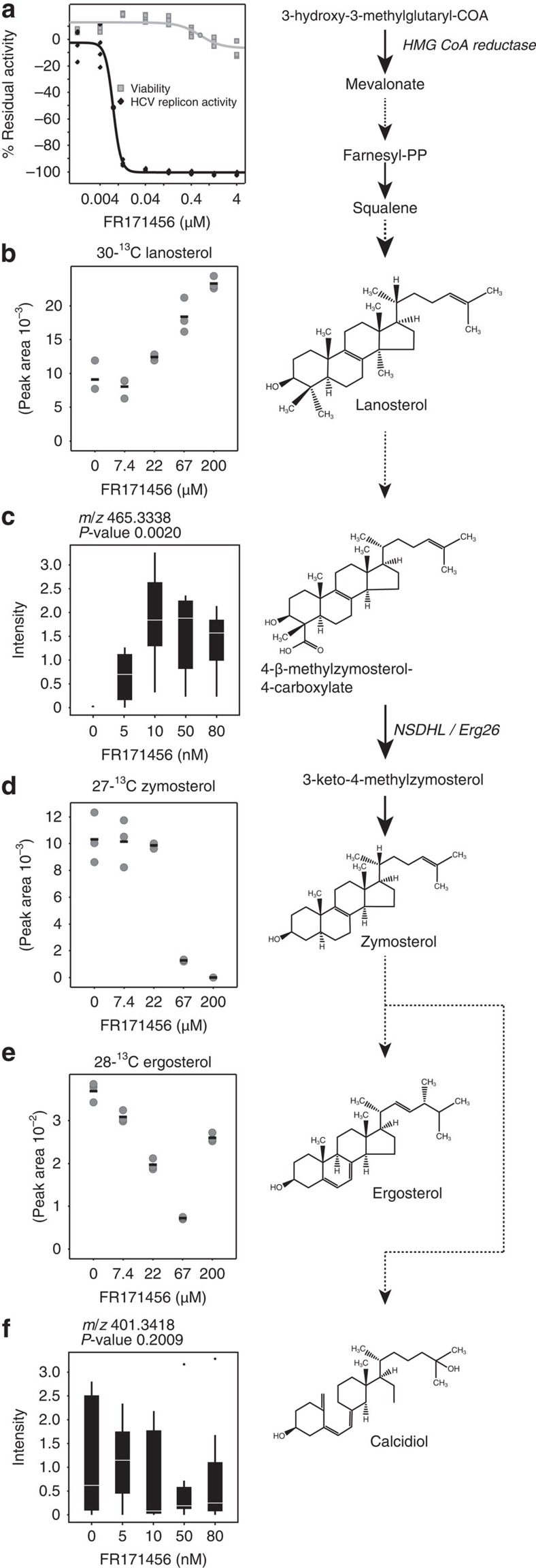
FR171456 inhibits HCV replication and alters the levels of multiple sterol pathway intermediates. (**a**) The effect of increasing doses of FR171456 on Huh-7 human cell proliferation (grey symbols) and the HCV replicon (black symbols) were measured and plotted as a dose-response curve. (**b**–**f**) The sterol pathway is shown highlighting selected products/substrates of pathway enzymes that could be detected as significantly altered in either Huh-7 cells treated for 48 h with increasing doses of FR171456 (**c**,**f**; black candlestick - bars and sticks represent 25/75th percentile and 1.58 times the interquartile range, respectively, from 15 separate measurements), or *C. albicans* cells treated with increasing doses of FR171456 for 14 h (**b**,**d** and **e**; grey circles and black bar represent the values and mean respectively from three biological replicates.

Bioactivity guided fractionation identified FR171456 with an IC_50_ of 6.3 nM in a Huh-7 cell-based assay that measures HCV replicon activity^16^. Although potent in the replicon assay the compound did not affect the proliferation of the replicon-carrying Huh-7 cells at concentrations up to 4 μM under these assay conditions (Fig. 1a). The compound did not affect the proliferation of two other mammalian cell lines, HepG2 and K562, except at very high concentration (80 and 36 μM, respectively). In a screen to profile compound activity against 503 cancer cell lines only five cell lines were sensitive to FR171456 at an *A*_max_ <50% and IC_50_ <5 μM (Supplementary Fig. 1)^18^.

FR171456 was previously identified as a compound that blocks cholesterol synthesis at or after the squalene synthesis step of the pathway^14^. Various stages in the lifecycle of HCV require a normally functioning cholesterol biosynthesis pathway^19^, suggesting that FR171456's effect on cholesterol synthesis is the reason why it scored in the HCV replicon assay. In an attempt to understand which sterol (and other) metabolites are altered by FR171456 a metabolite-profiling experiment was conducted using cells from the original replicon assay exposed to vehicle or four concentrations of FR171456 (Fig. 1c,f, Methods, supplementary Fig. 2). Peaks in the mass spectra with statistically significant differences in intensities between the high (80 nM) and low (0 nM) concentration samples were selected by *P* values <0.01 (Student's *t*-test, Bonferroni corrected) and ranked by fold changes. Metabolite identities were assigned to those peaks whose *m*/*z* ratio corresponds to the mass of known metabolites (Supplementary Table 1).

Analysis of the most significantly increased metabolite peaks in this set revealed one at m/z 465.3339 Da (Fig. 1c, Supplementary Table 1). This corresponds to the mass of the known substrate of NSDHL, 4-β-methylzymosterol-4-α-carboxylate^11^,^20^. The dose-dependent increase in intensities of this peak is consistent with inhibition of the NSDHL enzyme. A second peak at 467.3496 could represent 3-β-hydroxy 4β-methyl 5α-cholest-7-ene 4-α -carboxylate, that differs from 4-β-methylzymosterol-4-α-carboxylate by two hydrogens (Supplementary Table 1). This may represent an alternative reduced form of the substrate for NSDHL that would not normally be produced by these cells, but accumulates on blockage of the biosynthetic flux by FR171456 treatment. Reviewing this dataset for other known sterols revealed that calcidiol (*m*/*z* 401.3418), a cholesterol metabolite situated downstream of NSDHL, is decreased in a FR171456 dose-dependent manner, consistent with a reduction of cholesterol synthesis (Fig. 1f). No other sterol masses could be unequivocally assigned. Consistent with this data are the interpretation that FR171456 inhibits sterol synthesis between NSDHL and Calcidol synthase in Huh-7 cells.

Interestingly, three additional metabolites were identified as increasing in a dose-dependent manner. The peak at *m*/*z* 463.3183 may correspond to Pfaffic acid, but the other two masses, *m*/*z* 489.3314 and *m*/*z* 1198.8982 cannot be assigned. The accumulation of these peaks at doses that correspond well to the activity of the compound in the HCV replicon assay suggests that these metabolites are possibly linked to the cholesterol synthesis pathway, as this pathway has been implicated in replication of the HCV replicon^19^.

### FR171456 is antifungal and inhibits ergosterol synthesis

FR171456 demonstrated broad antifungal activity in a growth culture turbidity assay with standard minimum inhibitory concentration^21^,^22^, in the range of 9–144 μM with *Aspergillus terreus* as the most susceptible species (Supplementary Table 2). To assess whether FR171456 had effects on fungal ergosterol synthesis similar to those in mammalian cells (ergosterol is the fungal/plant equivalent of cholesterol), ergosterol pathway intermediates were measured following labelling of *C. albicans* with ^13^C-glucose, ^13^C-acetate and treatment with increasing doses of FR171456 (0–200 μM). The well-characterized Erg11p (Lanosterol 14-α demethylase) inhibitor fluconazole was used as a control (Supplementary Fig. 3). Consistent with its known activity, fluconazole caused an accumulation in ^13^C-labelled lanosterol and a decrease in both ^13^C-labelled zymosterol and ergosterol concentrations. FR171456 caused a dose-dependent reduction in zymosterol and ergosterol production, and an increase in lanosterol (Fig. 1b,d,e). At the highest tested concentration of FR171456, the amount of ^13^C-labelled ergosterol was increased compared with the vehicle control for reasons that are not clear. These data are consistent with the results from the mammalian cell metabolomics and previous work^14^ and suggests that FR171456 inhibits a target downstream of lanosterol which may be NSDHL/Erg26p.

To further evaluate the potential use of FR171456 as an anti-fungal, we assessed FR171456 activity on *R. oryzae* and *F. solani* (clinical pathogens) in combination with two clinical antifungal agents, Posaconazole and Amphotericin B. No synergy was detected with posaconazole on either microbe, nor was it detected with Amphotericin B on *R. oryzae* (Loewe synergy score <1 in all three cases, data not shown). However, a small but significant effect was observed when treating *R. oryzae* with a combination of Amphotericin B and FR171456 (Supplementary Fig. 4), with a Loewe synergy score of 2.7 (where anything >1 is synergistic^23^). Interestingly, at several doses of FR171456 that alone are relatively ineffective (6.67 and 20 μM), the amount of Amphotericin B required for maximal inhibition is halved.

### Genomic profiling identifies Erg26p as the FR171456 target

**Figure 2:**
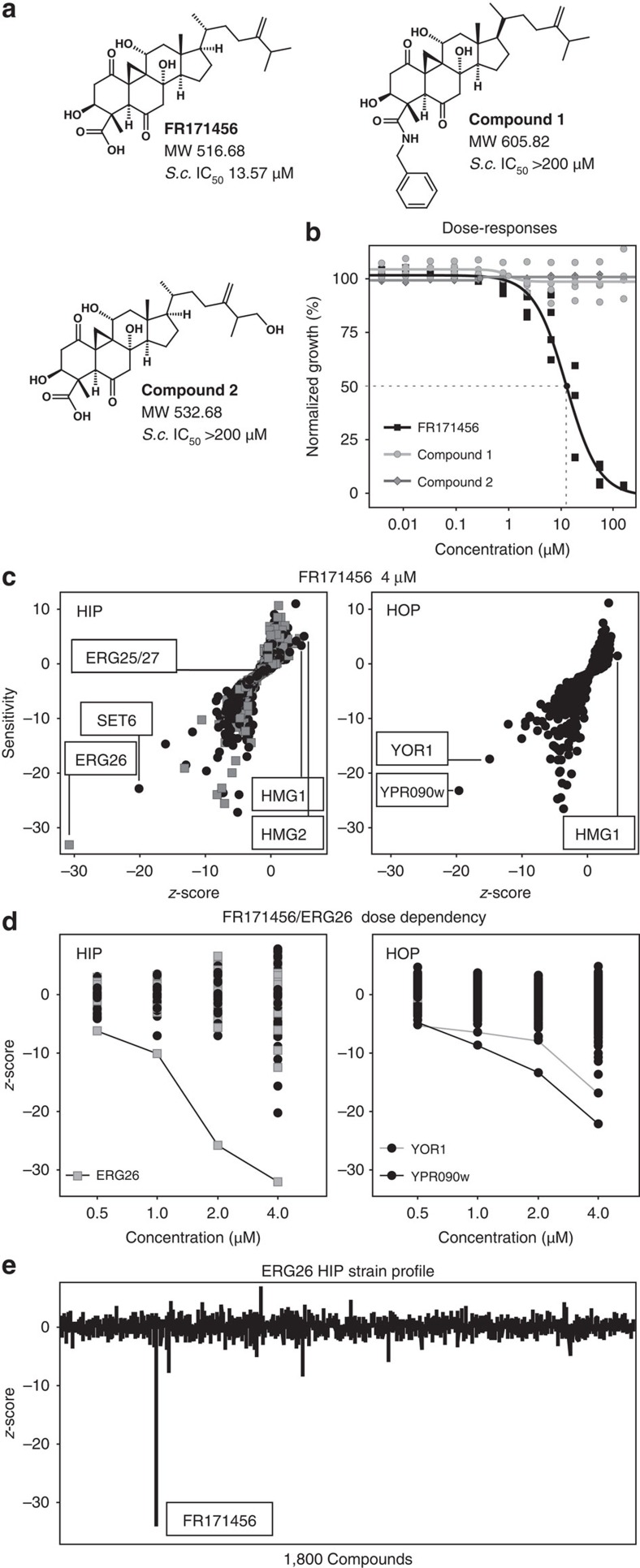
FR171456 targets Erg26p in *S. cerevisiae*. (**a**) Structures of FR171456, and two related triterpenes (compound 1, compound 2). (**b**) Duplicate 12-point Dose response curves of wild-type diploid yeast with three compounds in **a**. Strain BY4743 was grown for 14 h at 30 °C in YPD media with 2% DMSO containing a dilution series of FR171456: black; Compound-1: light grey circles, and Compound-2: light grey squares. *Y* axis: OD_600_ absorbance indicating yeast growth, *x* axis: compound concentrations (log10 μM). (**c**) Haploinsufficiency and homozygous profiling (HIP resp. HOP) for FR171456 at 4 μM. Each mutant strain in the pool is represented by a circle (non-essential genes) or a square (essential genes). The, *y* axis represents strain sensitivity and *x* axis represents *z*-score (specificity). In (**d**), the z-scores of the HIP pool strains (*y* axis) plotted against increasing doses of FR171456 (*x* axis; μM). (**e**) Relative compound sensitivity profile of the *erg26Δ/ERG26* strain across HIP experiments performed with 1,800 diverse small molecule yeast proliferation inhibitors.

FR171456 inhibited growth of *S. cerevisiae* (BY4743 strain) with an IC_50_ of 14 μM, but neither FR171456 derivative (Compound 1, Compound 2, see Methods) demonstrated any significant growth inhibition at concentrations up to 200 μM (Fig. 2a,b). Haploinsufficiency (HIP)/homozygous (HOP) profiling^24^ was performed using FR171456 at a concentration that would cause a 30% inhibition (IC_30_) of growth in a standard overnight culture (Fig. 2c). In the HIP profile, the ERG26 heterozygous strain (*erg26Δ/ERG26*), shows the most significant hypersensitivity to FR171456. In addition, the SET6 heterozygote is also significantly hypersensitive. The SET6 strain often scores when profiling compounds that inhibit ergosterol synthesis in *S. cerevisiae*^25^. Erg26p functions in a complex with Erg25p and Erg27p (ref. 10), but strains heterozygous for *ERG25* and *ERG27* were indistinguishable from wild type in the HIP profile. This may suggest that Erg26p is the limiting factor in the enzyme complex, and also suggests high specificity of FR171456 for Erg26p. In addition, HIP and HOP was repeated using different concentrations of FR171456, revealing altered sensitivity of the ERG26 strain in a dose-dependent manner (Fig. 2d).

Two homozygous diploid deletion strains demonstrated significant hypersensitivity to FR171456, YOR1 (*yor1Δ/yor1Δ*) and YPR090w (*ypr090wΔ/ypr090wΔ*). *YOR1* encodes a known multidrug resistance pump for which FR171456 is likely a substrate. *YPL090w* encodes an ORF of unknown function whose deletion confers sensitivity to miconazole, an ergosterol synthesis inhibitor, for reasons that are as yet unclear^26^. HMG-CoA reductase catalyses the conversion of HMG-CoA to mevalonate, the rate-limiting step in sterol biosynthesis. Strains heterozygous for either one of the two genes encoding the yeast HMGCR (*HMG1* and *HMG2* 27), and therefore carrying three out of four copies of HMGCR show weak but significant resistance to FR171456 (Fig. 2c). Furthermore, the HMG1 HOP strain carrying two from four copies of the HMGCR is also resistant (Fig. 2c). In these situations the effects of FR171456 are presumably partially remediated by a reduction in flux through the pathway, perhaps because an increase in sterol intermediate(s) between HMGCR and Erg26 is toxic.

Assessing the relative fitness of the ERG26 HIP strain across HIP profiles produced using 1,800 structurally unique compounds^24^ demonstrates that FR171456 is the only compound that has a significant effect on this strain (Fig. 2e). These results indicate that Erg26p is the major target of FR171456 in *S. cerevisiae*.

### FR171456-resistant *S. cerevisiae* Erg26p mutants

The isolation of yeast mutants that confer resistance has been used to identify the target of novel compounds and their binding pockets^28^,^29^. In a semi-biased approach to identify critical sites on Erg26p that potentially interact with FR171456, we randomly mutagenized a haploid yeast strain lacking two major multidrug-resistance pumps and selected FR171456-resistant colonies on solid, rich, media containing FR171456 at a concentration that prevented wild-type colony formation (50 μM). For 42 resistant clones, we sequenced only the *ERG26* coding region—from these, 35 had single nucleotide alterations within *ERG26*. These mutations cause single-amino-acid changes in any one of only eight amino acids within the coding region of *ERG26* gene (Supplementary Fig. 5, Supplementary Table 3). The other 7 resistant colonies were wild type for *ERG26* and were not pursued further.

**Figure 3:**
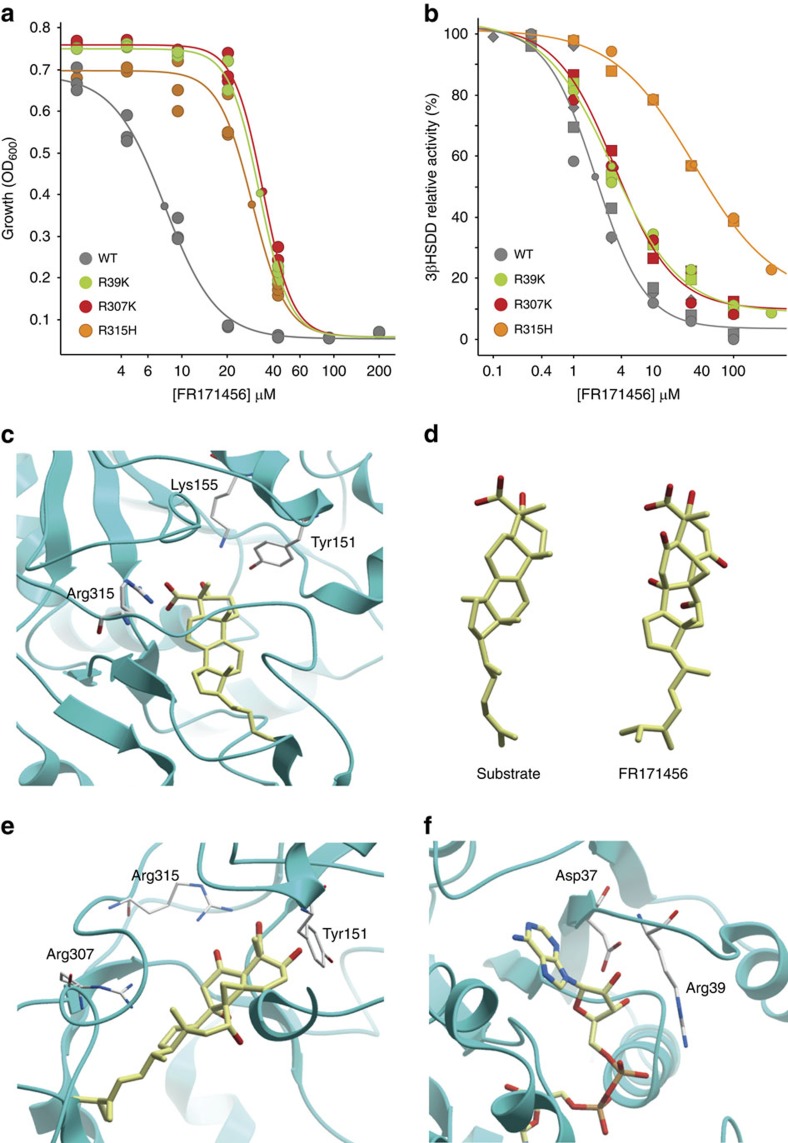
FR171456 inhibits Erg26p *in vitro* (**a**) Dose–response curve for yeast haploid strains with single copy of ERG26 carrying single point mutations conferring resistance to FR171456 (see also Supplementary Fig. 6). *Y* axis: OD_600_ absorbance indicating growth, *x* axis: compound concentrations, dose response performed in triplicate. (**b**) FR171456 inhibits *S. cereviasiae* Erg26p enzyme activity as demonstrated by microsome-based biochemical assay, dose response performed in triplicate (wt) or duplicate (mutants). *Y* axis: relative reaction velocity compared with *A. thaliana* 3BHSDD, *x* axis: FR171456 concentration. (**c**) Homology modeling of *S. cerevisiae* Erg26p with close up of active site with substrate and key interacting amino acids Tyr151, Lys155 and Arg315. (**d**) 3D structure of Erg26p substrate and FR171456 (see also Supplementary Fig. 9). (**e**) similar to (**c**) but with FR171456 docked, with Tyr151, Arg307 and Arg315 highlighted. Arg307Lys and Arg315His are changes giving resistance to FR171456 (**f**) Homology modelling of *S. cerevisiae* Erg26p with close up of NAD binding site with Arg37 and Arg39 highlighted. Arg39Lys mutants are FR171456 resistant.

To confirm that the observed *ERG26* mutations are dominant and sufficient to confer FR171456 resistance, but retain the essential function of Erg26p, we assessed the growth of *erg26Δ* haploid yeast expressing wild-type *ERG26* or one of seven mutant alleles inserted at another locus in rich liquid media. All these mutations caused an increase in the IC_50_ compared with the isogenic control (Fig. 3a, Supplementary Fig. 6). It was not possible to generate a haploid strain carrying a single copy of *ERG26* encoding the Gly90Ser mutation after multiple attempts. Thus seven of the eight mutations within the coding region of *ERG26* are both dominant for FR171456 resistance and retain sufficient enzymatic function to support life. This additional genetic data set confirms that Erg26p is the target of FR171456.

### FR171456 inhibits Erg26p enzymatic activity

Metabolic and genetic evidence suggests that Erg26p is the target of FR171456, presumably by directly inhibiting its enzymatic function. To test this hypothesis an *in vitro* microsomal Erg26p activity assay^30^ was used. Erg26p activity was directly assessed in the presence of increasing concentrations of FR171456 (10 nM–0.1 mM) in the presence of 150 μM carboxysterol substrate (Fig. 1c) and the cofactor NAD^+^ (0.5 mM) followed by GC-MS detection of the product 4α-methyl-cholest-8,24-dien-3-one. Figure. 3b shows that FR171456 strongly inhibits Erg26p activity *in vitro*. These results allowed the concentration of FR171456 required to reduce the reaction velocity to half (*I*_50_) to be determined: *I*_50_=2.2±0.5 μM.

*K*_m_ is an apparent dissociation constant for an enzyme–substrate complex and K*i* is the dissociation constant for an enzyme-inhibitor complex. The inhibition constant (K_i_ apparent) 31 value for Erg26p/FR171456 was 1.35±0.3 μM after fitting to a simple Michaelis-Menten mechanism inhibition equation. The *K*_m_ value of the carboxysterol substrate for the wild-type yeast Erg26p has been previously measured to be 550 μM (ref. 32). The *I*_50_/*K*_m_ ratio is used to compare the relative affinity towards the enzyme^31^, so FR171456 is a strong *in vitro* inhibitor of Erg26p with an approximate *I*_50_/*K*_m_ ratio of 4 × 10^−3^ for the carboxysterol substrate. Thus, this is the first reported *in vitro* enzyme activity that demonstrates that FR171456 has a high affinity for Erg26p and is a strong inhibitor of Erg26p.

### Erg26p inhibition requires the 4α-carboxyl group of FR171456

Previous work with *A. thaliana* 3βHSD/D (NSDHL) indicates that the formation of an Arg326-substrate carboxylate salt bridge is required for productive binding of the substrate in the plant enzyme active site^13^. This is in accordance with the strict substrate specificity of the 3bHSD/D that requires a free C-4 carboxyl group^12^. The Erg26p equivalent of *A. thaliana* Arg326 is Arg315. Substitution of the 4α-carboxylic group of FR171456 by a carbobenzylamido group (Compound-1) decreased inhibition by > 300 fold (*I*_50_>0.5 mM) confirming the requirement for the free C-4-carboxylic group for Erg26p inhibition by FR171456. This data suggests that this specific interaction is conserved between the plant and yeast enzymes.

### FR171456 docking into homology model of *S. cerevisiae* Erg26p

In the absence of a crystal structure for *S. cerevisiae* Erg26p, or its human and plant homologues, a homology model of the enzyme was built based on the crystal structure of *Pseudomonas aeruginosa* UDP-N-acetylglucosamine 4-epimerase complexed with UDP-N-acetylgalactosamine (PDB ID: 1SB8) (Supplementary Fig. 8a). The homology model also shows conserved structural domains with three other templates in Protein Data Bank (Supplementary Fig. 8b–d), but the 1SB8-derived model scored the best overall using multiple criteria (see Methods). To improve the accuracy of the model we determined the nuclear magnetic resonance (NMR) structure of the natural substrate following its purification from yeast inhibited with FR171456 (Methods, Fig. 3d, Supplementary Fig. 7).

The docked substrate and NAD^+^ cofactor interactions with the *S. cerevisiae* Erg26p active site shown by the model were consistent with those previously determined in the case of the plant *A. thaliana* 3βHSD/D (ref. 13; Fig 3c,f). These include the conserved catalytic Tyr151xxxLys155 motif proximal to the 3β-hydroxyl group of the carboxysterol, the Arg315 residue proximal to the 4α-carboxylic group of the substrate, and the Asp37 residue bridging the two hydroxyl groups of the adenine ribose of NAD^+^ in accordance with the NAD^+^ versus NADP^+^ cofactor specificity. Note that Erg26p Arg315 is equivalent to Arg326 in *A. thaliana*, and forms a similar interaction with the carboxylic group of the substrate.

The X-ray and NMR structures of FR171456 were solved, indicating significant similarity between the inhibitor and the substrate (Fig. 3d, Supplementary Fig. 9, Table 1). Taking this into account, and the significance of the inhibitor 4α-carboxylic group, FR171456 was docked in the carboxysterol binding pocket of *S. cerevisiae* Erg26p (Fig. 3e, Supplementary Fig. 10). The model suggests 3β-hydroxyl and 4α-carboxyl interactions with Tyr151 and Arg315, polar interactions between the 6-oxo group of FR171456 and Cys312, the 1-oxo group and Ser86, and hydrophobic interactions between Phe214 and the steroid nucleus of FR171456. Thus this model supports the hypothesis that FR171456 binds to Erg26p in the substrate-binding pocket.

### Modelling FR171456 resistance mutations on Erg26p

The mutations that conferred resistance against FR171456, but remain functional as measured by yeast proliferation, were mapped onto the *S. cerevisiae* homology model. According to this model FR171456-resistant mutations are located as follows: Arg315His preserves the capacity of this catalytic residue to make a salt bridge with the substrate carboxyl group, preserving the catalytic activity of *S. cerevisiae* Erg26p, but presumably reducing the binding of FR171456. In the case of the human enzyme NSDHL, Arg315 is substituted by His, demonstrating that this functionally conservative mutation still allows productive binding of the carboxysterol substrate. A second conservative mutation also located in the active-site pocket, Arg307Lys, is also likely to retain a viable catalytic activity, but could perturb the FR171456 interactions with the active site (Fig. 3e).

Two proline mutations, Pro144Leu and Pro147Leu, are not located in the active site, although Pro147 is proximal to the catalytic Tyr151 residue. Pro147 is conserved in *P. aeruginosa* UDP-*N*-acetylglucosamine 4-epimerase complexed with UDP-*N*-acetylgalactosamine (1SB8). This could play a role in the global architecture of the active site, and possibly in differential accessibility of the substrate and inhibitor to their binding domain. The Arg39Lys mutation is located in the active-site pocket (Fig. 3f). This functionally conservative mutation is proximal to the 2-adenosine ribose hydroxyl groups and diphosphate group of NAD^+^, and as such likely affects NAD^+^ binding and indirectly the interactions of its nicotinamide amido group with FR171456, possibly reducing its affinity.

Thus many of the FR171456 resistance mutations were mapped close to the NAD^+^ or the substrate-binding site. The presence of three active conservative mutations Arg39Lys, Arg307Lys, and Arg315His in proximity to NAD^+^ and substrate-binding site are consistent with FR171456 binding the substrate-binding pocket thus inhibiting the enzymatic activity. To address this more directly, we tested microsome extracts from the *erg26Δ* haploid yeast strains expressing the Erg26p wild type, Arg39Lys, Arg307His and Arg315His mutants in increasing doses of FR171456 to assess Erg26p mutant activity (Fig. 3b). Whilst Arg39Lys and Arg307His shift the *I*_50_ 2-fold, Arg315His confers a 20-fold shift in *I*_50_. This highlights the importance of the interaction between Arg315 and the carboxyl group of the substrate/inhibitor as one of the most critical in the Erg26p active site (Fig. 3c).

## Discussion

One of the central challenges facing chemical biologists is to ‘identify a small-molecule inhibitor for each individual function of all human proteins'^33^. In this study, FR171456 was reidentified due to its inhibitory activity in a phenotypic assay for HCV replicon, purified and its structure solved. Results from metabolomics, biochemistry, yeast genetics, and protein/inhibitor/substrate structure modelling, are all consistent with FR171456 specifically inhibiting yeast Erg26, and the mammalian homologue NSDHL, an enzyme distal to squalene synthase in the sterol biosynthesis pathway. This is the first identification of an inhibitor for Erg26p/NSDHL, and contributes to the collection of compounds targeting specific points/ nodes in the ergosterol/cholesterol biosynthesis pathway. The availability of a small molecule inhibitor of this node for the first time allows the inhibition of a node of sterol synthesis for which null mutations are unviable in yeast or mammalian systems. Such a tool compound will be useful to dissect the sterol pathway post-squalene in detail and suggests that NSDHL could be a drugable node^34^.

In addition to representing a specific and potent molecular probe for NSDHL function, this molecule demonstrates several lead-like properties, most significantly showing *in vivo* efficacy on the target pathway. Hatori *et al* treated rats at up to 10 mg kg^−1^ in short term experiments and showed a significant reduction in hepatic sterol synthesis even at doses as low as 0.1 mg kg^−1^, demonstrating a potent effect on the cholesterol pathway. Most importantly, these authors treated rabbits for 3 weeks, demonstrating a significant reduction in serum cholesterol already at the 0.001 mg kg^−1^ dose, with no adverse effects reported for this experiment even at doses up to 1 mg kg^−1^ (ref. 14). In addition, pharmacological profiling against a panel of 24 receptors/enzymes identified only one target with significant binding, the delta-opioid receptor, where an IC_50_ of 100 nM was observed in a radioligand competition assay. Thus FR171456 has reasonable chemical properties, efficacy and specificity *in vitro*, and efficacy *in vivo* with no adverse events reported in two rodent models, all key properties of a lead molecule.

It has been demonstrated previously that host lipid metabolism is important for the lifecycle of HCV for several reasons: (1) the low-density lipid (LDL) receptor is important for HCV entry; (2) the mevalonate pathway is required for HCV RNA replication; and (3) very low-density lipid (VLDL) is essential for co-secretion with virus particles^35^. The isolation of FR171456 in the HCV replicon assay provides further evidence that viral replication is modulated by host sterol and lipid metabolism^19^,^36^,^37^,^38^,^39^. Thus, FR17145 represents a potential starting point for novel anti HCV drugs, and NSDHL in turn a novel target.

The compound also exhibited reasonable antifungal activity against a wide spectrum of fungal species such as *S. cerevisiae, C. albicans, A. fumigatus* with minimum inhibitory concentration^21^,^22^ in a range from 4 to 64 μg ml^−1^ (9–144 μM). Although these values do not make FR171456 a particularly potent small molecule, mammalian cell proliferation is unaffected by FR171456 in almost 500 cell lines tested *in vitro*^18^. In addition, in the original report describing FR171456, rabbits were treated with efficacious doses for 14 days with no reported side effects^14^. Taken together these data raise the hope that there may be a reasonable therapeutic window *in vivo* for compounds that inhibit this node in the sterol synthesis pathway.

Mutations in the NSDHL gene cause the human syndrome CHILD (congenital hemidysplasia with ichthyosiform nevus and limb defects), a dominant X-linked, male lethal disorder^40^. In recessive heterozygote males, the syndrome causes unilateral ichthyosiform skin lesions and limb reduction defects—known as CK syndrome^41^. For these pathologies, it has been suggested that not only cholesterol deficiency, but also accumulation of methyl sterols and carboxysterols causes disease^42^. Using FR171456 to cause the accumulation of upstream intermediates in relevant model systems should help to clarify this hypothesis. Indeed, the Huh7 cell metabolomics performed here demonstrated the accumulation of known and novel molecules in a dose-dependent manner, and the elucidation of the novel molecule structures may suggest mechanisms whereby NSDHL inhibition causes toxic intermediate accumulation.

## Methods

### Purification of FR171456

A strain of *Monodictys* Sp. (from the Novartis strain collection) was cultured in medium MP3–01.00 (20 g l^−1^ defatted soya, 20 g l^−1^ D-Mannitol, 1 ml active solution Nr 1901, which contained 4 g l^−1^ ZnSO_4_.7H_2_O), 0.1 g l^−1^ H_3_BO_3_, 5 g l^−1^ FeSO_4_.7H_2_O, 0.005 g l^−1^ KI),1 g l^−1^ CoCl_2_, 0.2 g l^−1^ CuSO_4_. 5H_2_O, 2 g l^−1^ MnCl_2_.4H_2_O, 1 g l^−1^ (NH_4_)_6_(Mo_7_O_2_)_4_.4H_2_O. The culture was started with a 2.5% inoculum of a preculture which had been grown for four days from a cryovial of the strain. A 2-l culture was then cultured for 6 days at 24 °C at 200 r.p.m. The broth was filtrated through Celite. The filter cake containing the mycelium was extracted with ethyl acetate resulting in 0.5 g of extract. The complete ethyl acetate extract was separated on a reversed phase column (50 × 200 mm column packed with Merck Lichrospher RP18, 12-μm particle size). The elution gradient was from 30% acetonitrile in water rising to 100% within 60 min, the mobile phase contained 0.01% trifluoroacetic acid (TFA). Combined fractions resulted in 9.9 mg of purified FR171476.

The structure of FR171476 was determined by infrared spectroscopy, mass spectroscopy (MS) and NMR spectroscopy. The structure has been previously reported^14^. FR171456 can be described chemically as (1R,2aR,3R,5aR,5bS,7aR,8S,9S,11aS,12aR)-1,5b,9-trihydroxy-2a,8-dimethyl-3-((R)-6-methyl-5-methyleneheptan-2-yl)-7,11-dioxohexadecahydrocyclopenta[a]cyclopropa[e]-phenanthrene-8-carboxylic acid.

A sample was analysed by 1H NMR (600 MHz, dimethyl sulfoxide (DMSO)-d6) *δ* p.p.m. 0.68 (s, 3 H) 0.89 (d, *J*=6.59 Hz, 3 H) 0.98–1.00 (m, 6 H) 1.00 (s, 3 H) 1.07–1.16 (m, 2 H) 1.17–1.35 (m, 4 H) 1.37 (d, *J*=5.85 Hz, 1 H) 1.44–1.55 (m, 1 H) 1.64 (q, *J*=9.03 Hz, 1 H) 1.78–1.91 (m, 3 H) 1.93–1.99 (m, 1 H) 2.02–2.11 (m, 2 H) 2.14–2.25 (m, 2 H) 2.32 (dd, *J*=16.83, 8.78 Hz, 1 H) 2.51 (dd, *J*=12.44, 7.32 Hz, 1 H) 2.83 (dd, *J*=16.83, 7.32 Hz, 1 H) 4.15 (*t*, *J*=8.05 Hz, 1 H) 4.21 (s, 1 H) 4.38 (br. s., 1 H) 4.51 (d, *J*=6.59 Hz, 1 H) 4.64 (s, 1 H) 4.70 (s, 1 H) 4.97 (br. s., 1 H) 5.23 (br. s., 1 H).

The absolute configuration of FR171456 has been identified by high resolution X-ray structure determination. The structure of the compound is shown in Fig. 3d and Supplementary Fig. 9. X-ray data for FR171456 is available in Table 1. This is the first report of the absolute 3D structure of this compound.

### Synthesis of compound 1

Compound 1[(1R,2aR,3R,5aR,5bS,7aR,8S,9S,11aS,12aR)-N-benzyl-1,5b,9-trihydroxy-2a,8-dimethyl-3-((R)-6-methyl-5-methyleneheptan-2-yl)-7,11-dioxohexadecahydrocyclopenta[a]cyclopropa[e]phenanthrene-8-carboxamide] was prepared as follows. To a solution of FR171456 (30 mg, 0.058 mmol), benzylamine (4.45 μl, 0.04 mmol), 1-hydroxy-7-azabenzotriazole (HOAt; 120 μl, 0.06 mmol) in 2.5 ml DMF, EDC.HCl (12.6 mg, 0.064 mmol) was added at room temperature. The reaction mixture was stirred for 2.5 h at room temperature. Since only partial conversion was observed, additional benzylamine (4.45 μl, 0.04 mmol), HOAt (120 μl, 0.06 mmol) and EDC.HCl (12.6 mg, 0.064 mmol) were added to the reaction. After 16 h at room temperature the reaction mixture was diluted in 20 ml of ethyl acetate and 5 ml of water. The organic phase was extracted with saturated NaHCO_3_ (2 × 5 ml) and brine (2 × 5 ml), dried over Na_2_SO_4_, and subsequently evaporated under reduced pressure. The crude product was separated by RP-HPLC on Luna C18 stationary phase and a gradient of the eluents (water/0.1% formic acid and acetonitrile/0.1% formic acid). Fractions containing compound-1 were combined, dried under reduced pressure and subsequently lyophilized to yield 9.4 mg (26.7%) of compound-1. Agilent 1100 LCMS analysis, MS+*m*/*z*=606.31. NMR data were recorded at 26 °C on a Bruker AV-I-600 or AV-III-600 spectrometer, using a 1.7 mm TXI cryoprobe. ^1^H NMR (600 MHz, DMSO-*d*_6_): 1H NMR (600 MHz, DMSO-d6) *δ* p.p.m. 0.69 (s, 3 H) 0.90 (d, *J*=5.85 Hz, 3 H) 1.00 (dd, *J*=6.50 Hz, 6 H) 1.13 (d, *J*=5.85 Hz, 2 H) 1.15 (s, 3 H) 1.17–1.36 (m, 4 H) 1.39 (d, *J*=5.85 Hz, 1 H) 1.45–1.57 (m, 1 H) 1.64 (q, *J*=9.51 Hz, 1 H) 1.84 (d, *J*=14.60 Hz, 1 H) 1.86–1.92 (m, 2 H) 1.97 (dd, *J*=14.64, 6.59 Hz, 1 H) 2.02 (d, *J*=19.03 Hz, 1 H) 2.05–2.11 (m, 1 H) 2.16 (d, *J*=18.30 Hz, 1 H) 2.18–2.26 (m, 1 H) 2.35 (dd, *J*=16.47, 8.42 Hz, 1 H) 2.45–2.55 (m, 1 H) 2.82 (dd, *J*=16.83, 6.59 Hz, 1 H) 4.11 (t, *J*=7.68 Hz, 1 H) 4.28 (dd, *J*=15.37, 5.85 Hz, 1 H) 4.41 (s, 2 H) 4.44 (dd, *J*=15.40, 5.90 Hz, 1 H) 4.47 (d, *J*=6.59 Hz, 1 H) 4.66 (s, 1 H) 4.72 (s, 1 H) 5.01 (br. s., 1 H) 5.20 (br. s., 1 H) 7.18–7.22 (m, 1 H) 7.28 (t, *J*=7.68 Hz, 2 H) 7.40 (d, *J*=7.32 Hz, 2 H) 7.87 (t, *J*=5.85 Hz, 1 H)

### Synthesis of compound 2

Compound 2[(1R,2aR,3R,5aR,5bS,7aR,8S,9S,11aS,12aR)-1,5b,9-trihydroxy-3-((2R)-7-hydroxy-6-methyl-5-methyleneheptan-2-yl)-2a,8-dimethyl-7,11-dioxohexadecahydrocyclopenta[a]cyclopropa[e]phenanthrene-8-carboxylic acid] was prepared as follows. The biotransformation of FR171456 with the strain Nocardioides simplex ATCC 6946 was performed in 0.5-l flasks each containing 95 ml liquid medium of 4 g/l D-(+)-glucose, 10 g l^−1^ malt extract, 4 g l^−1^ yeast extract and distilled water (pH 7,5). Each flask of the main culture was inoculated with 5 ml of the preculture and incubated for 2 days at 28 °C, 220 r.p.m. Solutions of FR1714556 (2.2 mg, mmol) in 4.4 ml methanol and 10 ml of 1 M KH_2_PO_4_ were added to each of the 11 flasks. After incubation for three days at 28 °C/180 r.p.m. the fermentations were stopped by adding 100 ml isopropanol to each culture. The solvents were evaporated under reduced pressure and the remaining broth was lyophilized. The dry powder was suspended in water and extracted with ethyl acetate. The organic phase was dried over Na_2_SO_4_ and evaporated under reduced pressure, yielding 405 mg of crude product. It contained the desired product based on HPLC analysis and no starting material was detected. The crude product was separated by preparative reverse phase chromatography using an Ascentis phenyl stationary phase and a gradient of the eluents (water/0.1% formic acid and acetonitrile/1% water/0.1% formic acid). Fractions containing compound-2 were combined, dried under reduced pressure and subsequently lyophilized. The sample was separated again using the same method yielding 5 mg of compound-2. Agilent 1100 LCMS analysis, MS+ m/z=479.3, fragmentation of parent ion observed. 1H NMR (600 MHz, DMSO-d6) *δ* p.p.m. 0.66 (s, 3 H) 0.87 (d, *J*=6.24 Hz, 3 H) 0.96 (d, *J*=6.60 Hz, 3 H) 0.98 (s, 3 H) 1.04–1.12 (m, 1 H) 1.15 (d, *J*=5.87 Hz, 1 H) 1.16–1.27 (m, 3 H) 1.28–1.33 (m, 1 H) 1.35 (d, *J*=5.14 Hz, 1 H) 1.44–1.54 (m, 1 H) 1.57–1.65 (m, 1 H) 1.76–1.88 (m, 3 H) 1.94 (dd, *J*=14.67, 6.60 Hz, 1 H) 2.00–2.08 (m, 2 H) 2.13 (dd, *J*=12.84, 6.97 Hz, 1 H) 2.18 (dd, *J*=19.07, 2.57 Hz, 1 H) 2.30 (dd, *J*=17.24, 8.80 Hz, 1 H) 2.44–2.53 (m, 1 H) 2.80 (dd, *J*=17.24, 7.34 Hz, 1 H) 3.18 (ddd, *J*=10.27, 7.70, 5.50 Hz, 1 H) 3.41 (dt, *J*=10.36, 5.27 Hz, 1 H) 4.12 (t, *J*=7.89 Hz, 1 H) 4.19 (s, 1 H) 4.36 (d, *J*=2.57 Hz, 1 H) 4.43 (t, *J*=5.32 Hz, 1 H) 4.48 (dd, *J*=6.24, 2.93 Hz, 1 H) 4.67 (s, 1 H) 4.69 (s, 1 H) 4.97 (d, *J*=2.93 Hz, 1 H) 5.19 (br. s, 1 H) 11.89 (br. s, 1 H). NMR data were recorded at 26 °C on a Bruker AV-I-600 or AV-III-600 spectrometer, using a 1.7 mm TXI cryoprobe.

### Isolation of compound 3 by Erg26p substrate purification

In the fermentation experiment, a single yeast colony was grown in 10 ml YP with galactose (2%) & Raffinose (1%) for 5 h (OD 1.0) at 30 °C. An inoculum (70 μl) from the same culture was used to inoculate 400 ml YP-gal (2%)+Raffinose (1%). This culture was then grown overnight at 30 °C. The OD at this point was 0.7. This culture was used to seed 20-l fermenter maintained at 30 °C. When OD in the fermenter was 0.7, then 1466, nM (IC_80_) of FR171456 was added to the fermenter. The cell pellets (3.8 g) were harvested after 7 h. Erg26p substrate (1.95 mg) was obtained by lipid extraction with ethylacetate and methanol.

The extraction protocol included suspension of collected yeast cells in 250 ml methanol: water mixture (9:1). The extract was defatted by extraction with 250 ml cyclohexane yielding 2.8 g defatted extract. The defatted extract was then subjected to chromatograph with a Sephadex LH20 (5 × 100 cm) column using methanol as the solvent. The fraction (205 mg) containing the desired compound was then further purified using reversed phase HPLC with a 30 × 250 mm Sunfire RP18 column (5 μm). The column was developed using the following solvents (A: 0.03% formic acid, B: acetonitrile containing 0.03% formic acid). The column was first washed with a solution of 70% B for 5 min followed by a gradient from 70% B to 100% B in 25 min yielding in 1.1 mg desired substrate.

1H-NMR was used to determine the structure of the substrate. The parameters for the instrument were: 660.13 MHz frequency, PROBHD: 1.7 MM CPTCI1, DMSO solvent, PULPPROG ZG30, TD 65536. The NMR data for the compound:^1^H NMR (600 MHz, DMSO-*d*_6_) *δ* p.p.m. 0.55 (s, 3 H) 0.91 (d, *J*=6.60 Hz, 3 H) 0.92 (s, 3 H) 0.95 (s, 3 H) 0.97–1.03 (m, 1 H) 1.09–1.16 (m, 1 H) 1.21–1.25 (m, 3 H) 1.27–1.31 (m, 3 H) 1.31–1.39 (m, 2 H) 1.43–1.54 (m, 4 H) 1.55 (s, 3 H) 1.63 (s, 3 H) 1.63–1.67 (m, 1 H) 1.73 (dd, *J*=12.62, 0.91 Hz, 1 H) 1.78–1.87 (m, 3 H) 1.88–2.06 (m, 5 H) 3.70 (dd, *v*10.98, 3.66 Hz, 1 H) 5.06 (t, *J*=6.59 Hz, 1 H).

### HCV replicon inhibitor screening

The inhibitor screening was performed in 1,536-well plate with 3,000 cells per well, using Huh-7 cells containing the HCV replicon subgenomic genotype 1b, maintained in DMEM, supplemented with (1 × ) L-glutamine (L-Glue), 1 × non-essential amino acids, 10% heat-inactivated fetal bovine serum (FBS), and 1 mg ml^–1^ G418, and incubated with compounds for 48 h at 37 °C, 5% CO_2_. Compounds or microbial extracts were diluted in 90% DMSO and then diluted in culture media so that the addition of 10 nl of dilutant to the cells resulted in a final DMSO concentration of 0.18%. Following compound incubation, Resazurin solution (10 × : 1 μg μl^–1^ Resazurin in DMSO) diluted to 1 × was then added to a volume of 0.5 μl to each well and incubated for further 3 h at 37 °C, 5% CO_2_ to monitor cell viability. Subsequently 15 μl, diluted 1:2 from stock of Steady- Glo (Promega) was added and the luminescence from the HCV replicon was monitored using the Envision (Envision US Lum, using 0.1 s per well measurement time).

### Yeast strains and plasmids

BY4741 a *pdr5::kanMX snq2::kanMX* was constructed using standard techniques using strains obtained from Invitrogen (Cat no: 95400.BY4741); the heterozygous and homozygous deletion collections were also obtained from Invitrogen. pRS416 is a CEN URA3 shuttle vector. All cloning was performed using standard molecular biology techniques^43^.

For ^13^C- labelling studies, the wild type *C. albicans* strain SC5314 was obtained from ATCC (Manassas, VA) and was cultured at 30 °C in YPD broth (1% yeast extract, 2% peptone, 2% dextrose) or Synthetic Medium (3% glycerol, 0.1% glucose, 65 mM NH_4_Cl, 1.7 mM NaCl, 7 mM KH_2_PO_4_, 1 mM CaCl_2_, 0.5 mM MgSO_4_).

### Mutagenesis

For EMS mutagenesis experiments, 1 × 10^8^ cells were plated on one standard YPD agar plate containing 50 μM FR171456 in 2% DMSO v/v following a standard EMS mutagenesis protocol. After 2 days incubation at 30 °C 48 representative colonies were picked and retested in an overnight liquid assay for growth in YPD with or without 50 μM FR171456 in 2% DMSO v/v. Genomic DNA was prepared from 42 resistant clones (+ one wild type) and for each the entire *ERG26* gene was sequenced following PCR amplification with high-fidelity polymerase. These mutations were then cloned with their native promoter into BYintURA and then transformed into BY4743 *erg26::kanMX/ERG26* diploid yeast strain and processed to create haploid strains carrying the BYIntURA3 vector with wild type or mutant ERG26 in the *erg26::KanMX* background. The FR171456 dose−response assay for the mutants was performed in a 96-well plate format as described 24.

### Mass spectrometric analysis for global metabolomics profiling

Samples of cell pellets containing 8 × 10^6^ cells each were lysed and extracted with 300 μl cold methanol (−80 °C) in ultrasonic bath for 10 min and subsequently agitated for 5 min before centrifugation at 10,000 *g* for 5 min at 4 °C. The supernatants were removed and the remaining pellets were again extracted twice. The supernatants from three extractions were then combined and dried using a SpeedVac (Savant, Fisher Scientific Inc., USA) for 2 h without heating. The residues were resuspended in 200 μl of 80% MeOH and centrifuged to remove any non-dissolvable particles. Finally, 10 μl of the supernatant was then diluted by factor 100 in MeOH/H_2_O (1:1) for mass spectrometric analysis.

The diluted sample (100 μl) was infused directly into a LTQ-Orbitrap mass spectrometer (Thermo Fisher Scientific, USA) using a HTC PAL autosampler (CTC Analytics, Zwingen, Switzerland) at a flow rate of 18 μl min^–1^. A make-up flow, consisting of 70% ethanol and 0.1% formic acid, was delivered through a T-piece at 15 μl min^–1^. Electrospray ionization and selected monitoring scan were applied. The total mass range *m*/*z* 100–2,000 was segmented into 48 scan events with a width of mass window *m*/*z* 50 each. Mass resolution at *m*/*z* 400 was 100,000.

Each sample was measured in triplicate in positive ionization mode in random order. For quality and instrument stability control, the samples were interspersed with test mix infusion and pooled samples. Blank spectra were also recorded and the observed mass peaks of solvent and impurities were then suppressed in subsequent analysis.

The measured raw spectra were imported with in-house software written in the Igor package (Wavemetrics Inc.) for alignment, peak detection, and integral normalization. The table of exact peak masses and intensities (Supplementary Data, file Supplementary Dataset 1) were exported into the statistical software R, version 2.15.1, for subsequent univariate statistics and pathway analysis. For peak identification, the KEGG database was searched for matches of the observed peaks with endogenous metabolite entries. Metabolite masses with adducts of H^+^, Na^+^, or K^+^ were considered a hit and possible identity of a peak if the difference between its exact monoisotopic mass and the observed mass plus adducts was below 3 p.p.m.

### Focused metabolomics in *C. albicans*

Approximately 1.25 × 10^8^ *C. albicans* cells from a saturated YPD culture were inoculated into 100 ml Synthetic Medium (SM) and grown at 30 °C to an OD of 1.0. Cells were washed once in modified SM (25 mM KPO_4_ pH 7.0, with glucose omitted) and suspended to 5 OD_600_ units per ml in modified SM.

Labelling reactions were performed in 12-ml glass tubes with PTFE-lined caps (Corning) and containing 1 ml cell suspension (∼6 × 10^7^ cells), 10 μl DMSO or 10 μl compound dissolved in DMSO, 12 μl 1M D-Glucose (U^-13^C6 glucose), and 10 μl 1 M (1,2^-13^C2) sodium acetate (Cambridge Isotope Laboratories, Inc). Before LC-MS analysis, dried lipid extracts were dissolved in 500 μl methanol, vortexed and sonicated for 10 min.

Squalene, ergosterol, lanosterol and zymosterol were quantified using the tandem mass spectrometer API4000 triple quadrupole (AB Sciex) with atmospheric pressure chemical ionization (APCI), interfaced to an Agilent LC.

### Labelling reaction and lipid quantification

Cells (∼6 × 10^7^) and compounds were preincubated 15 min at 30 °C (without shaking) before the addition of labelled substrates. Labelling was performed at 30 °C with shaking for 4 h. Reactions were stopped by adding 1 ml 30% KOH, 1 ml 100% ethanol, and 0.2 ml 2% pyrogallol. Samples were mixed and then heated at 80 °C for 30 min, then cooled to room temperature. Non-saponifiable lipids were extracted two times in petroleum ether, washed with water, dried under nitrogen gas and stored at −80 °C.

For compound calibration, 2 μM solutions in 0.1% formic acid were directly infused into the mass spectrometer. Ergosterol, lanosterol and zymosterol undergo dehydration and their molecular species are detected as [M+H-H_2_O]^+^ while squalene is detected as [M+H]^+^. Supplementary Table 4 lists the multiple reaction monitoring parameters of the sterols and squalene, as well as the ^13^C incorporated metabolites. The mass spectrometer source parameters are the following: CUR=40, GS1=60, NC=4.5. TEM=450 CAD=4. A mobile phase of 0.2% formic acid in methanol and isocratic run at 0.4 ml min^−1^ was employed to separate the metabolites on a Waters XBridge C18 3.5 μm 2.1 × 50 mm column. The run time was 4.5 min.

### Metabolic Profiling for substrate accumulation in *S. cerevisiae*

Lipid extracts from yeast cells were prepared as described as above. Ergosterol and 4α-methylzymosterol-4-carboxylic acid were analysed on an UltiMate 3000 RS LC (Thermo Fisher Scientific, Waltham, MA, USA) coupled to a 4000 QTRAP (AB Sciex, Foster City, CA, USA), which was equipped with a ESI-Turbo Spray ion source (AB Sciex).

Chromatography was carried out on a Waters Acquity UPLC BEH C18, 1.7 μm, 1 × 50 mm column (Waters, Milford, MA, USA) with a flow rate of 200 μl min^−1^ and a gradient from 30% A (H_2_O with 0.1% (v/v) formic acid) and 70% B (acetonitrile with 0.1% (v/v) formic acid) to 2% A and 98% B in 3.5 min. Solvent composition remained for 4 min at 2% A and 98% B before the column was reconditioned to 30% A and 70% B. The total runtime for one analysis was 12 min. To reduce ion source contaminations, a switch valve was used to direct the flow to waste for the first 3.5 min and the last 5.5 min of the LC method.

Ion source parameters and compound-dependent instrument parameters were optimized by infusing pure standard solutions of ergosterol and 4α-methylzymosterol-4-carboxylic acid, respectively. Ergosterol was detected in positive ion mode by selected reaction monitoring (SRM) using the mass transitions 379.3 [M-H_2_O+H]^+^ → 69.2 and 379.3 → 83.1 and 4α-methylzymosterol-4-carboxylic acid was detected using the mass transitions 425.3 [M-H_2_O+H]^+^ → 95.1, 425.3 → 109.1 and 425.3 → 159.1. All mass transitions were monitored for 65 msec per SRM scan with a unit/unit resolution for Q1/Q3. Analyst 1.5.1 (AB Sciex) was used for data analysis and peaks were smoothed with the Savitzky-Golay algorithm with a smoothing half width of 3 points.

### HIP–HOP assay

The HIP assay was performed in 24-well plates (Greiner 662102), with 1.6 ml per well YPD. Experimental compounds were tested at *n*=2 within the same plate at or close to their IC_30_ concentration. Each plate contained two no drug controls, one positive control (Benomyl), 10 experimental compounds in duplicates and one contamination control that received no cells. A standard experiment was 4 plates per 40 experimental compounds processed robotically without human intervention. YPD per compound filled wells were inoculated with ∼250 yeast cells per strain (100 μl of a 1.5-OD_600_ per ml culture) from an overnight log phase preculture to start the experiment (Supplementary Fig. 2). The plates were pipetted with a standard 96 pipettor head by providing tip boxes preconfigured with a special tip pattern. Plates were incubated for 16 h in a robotic shaking incubator at 30 °C/550 r.p.m. allowing for ∼5 doublings. ∼250 yeast cells per strain (120 μl of a 1.2-OD_600_ per ml culture) were subsequently transferred into a preconfigured 24-well plate that was stored in a robotic plate reservoir at 4 °C until 30 min prior to its use where it was prewarmed to 30 °C. Once inoculated the new plate was incubated at 30 °C/550 r.p.m. to allow the next five yeast generations (generation 6–10) and the plate containing the first five doubling cultures was stored at 4 °C. This procedure was repeated two more times until the final plate containing the yeast with ∼20 generations were stored at 4 °C.

The HOP assay was performed similar to the HIP experiment but the duration was reduced to approximately five doublings and no dilutions were necessary. Before the experiment, aliquots of the HOP pool were thawed and recovered for 3 h in YPD. The robotic system inoculated the wells prefilled with YPD and compound at the onset of the experiment with ∼320 yeast cells/strain (110 μl of a 1.50-OD_600_ per ml culture). Plates were incubated for 16 h in a robotic shaking incubator at 30 °C/550 r.p.m. allowing for approximately five doublings and where then stored at 4 °C.

By applying a fixed time per dilution scheme to the HIP–HOP assay and diluting entire plates at fixed times we have simplified the procedure and managed to get optimal throughput. Careful IC_30_ determination and compound potency normalization resulted in very homogenous growth behaviours of the cultures in the 24 wells per plate. After each HIP–HOP experiment, growth curves recorded during the experiment were analysed.

*gDNA extraction, TAG amplification and hybridization*. An aliquot of 5 OD_600_ units of yeast per well from the HIP and HOP experiments were arrayed in 96-well plates, spun and the supernatant discarded. gDNA extraction was performed using the ChargeSwitch kit (Invitrogen #18000) in a partially automated process. One hundred and fifty microlitres per well of Zymolyase buffer (2 U Zymolyase, 50 ng RNase A, in 20 mM DTT and 20 mM Tris pH 7.5) were added and the cells were incubated at 37 °C/700 r.p.m. for 45 min. Three hundred microlitres per well lysis buffer (L18, Invitrogen) was added and the plate incubated at 56 °C/ 700 r.p.m. for 30 min. Two hundred microlitres per well ice-cold precipitation buffer (N2, Invitrogen) was added and the precipitate pelleted by centrifugation at 4 °C. The supernatant was saved to a new deep well plate (AB-0932, Abgene) suitable to lock onto the Invitrogen MagnaRack magnet and 40 μl per well of predispersed ChargeSwitch magnetic beads were added. The plate was incubated at room temperature for 5 min followed by incubation on the MagnaRack for 5 min to pellet the beads. All supernatant was carefully aspirated. The deep well plate was removed from the MagnaRack and 500 μl per well wash buffer (W12, Invitrogen) were added and mixed to disperse the beads. The washing was repeated three times and the wash buffer completely removed. Finally, 70 μl of elution buffer (E6, Invitrogen) were added. The beads were resuspended by mixing and the plate incubated for 10 min at room temperature. The beads were pelleted for one last time by incubation on the magnet for 5 min and the supernatant (containing the gDNA) was transferred to a new plate. The TAG PCR amplification and GenFlex Tag16K v2 hybridization protocol was used as described^24^.

*Processing of TAG16K v2 data*. The raw probe intensity values of the CEL are summarized and normalized to tag intensities. Tags with low intensity values in control samples are removed by computing an intensity value threshold based on the comparison of the correlation between the logarithmic intensity ratios for uptags and downtags across different intensity ranges. The tag intensities are then averaged to obtain a strain intensity value. To measure the relative abundance of each strain with respect to the averages of the control samples we compute *MAD logarithmic* (*MADL*) scores for each compound/concentration combination. If we denote the logarithm of the ratio of the average intensity of the compound samples over the average intensity of the control samples as *r*_*L*_, then the MADL *s*_*L*_ score is given as (*r*_*L*_–med(*r*_*L*_))/MAD(*r*_*L*_) where the median and MAD are computed over all strains in one sample. MADL scores can be viewed as robustly computed experiment-wise *z*-scores. We also compute the *t*-test *P* value, *P*, between the two replicates for a compound and the four to eight control replicates as a measure of the variability of the compound and control sample intensities across the experiment. The final (adjusted) score *a*_*L*_ is decreased for highly variable strains and computed as:


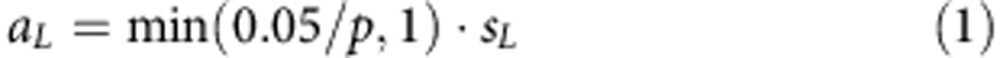


Then, we compute gene-wise *z*-scores (across all experiments) which are based on a robust parametric estimation of gene variability allowing for up to 15% outliers. To do this we consider the adjusted MADL scores (*a*_1_,..., *a*_*n*_) of a strain over *n* experiments. Usually, the z-score transformation of score *a*_*i*_ is defined as 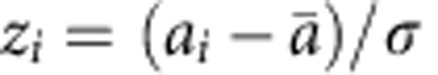 where 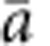 is the mean and *σ* the s.d. of the values *a*_*i*_. However, due to the special nature of the HIP–HOP data it is advantageous to introduce a number of changes to the computation of the adjusted MADL *z*-score transformation. Since an (adjusted) MADL score of zero indicates a relative growth rate of a strain in the compound treated sample that is equal to the relative growth rate of the strain in the untreated control, we set 
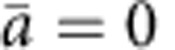
, for all strains. What remains is to estimate *σ* as a measure of the variability of the strain which is described in the following.

In order to avoid biasing the computation of the s.d. of a strain profile we allow at most five entries of the same compound/concentration combination in the data set. The samples of the sixth or greater occurance are discarded. We normalize the positive and negative scores separately; that is, we treat the positive and the negative scores as the halves of two separate distributions (artificially creating two perfectly symmetric distributions in this way). In the following we only consider the negative scores. Let the profile of the negative scores of a gene be (*s*_1_,..., *s*_*n*_) where we assume that the scores are sorted in ascending order. We assume that the scores follow a normal distribution *N*(*μ,σ*). Note that since the distribution is perfectly symmetric around 0, *μ*=0; moreover, for each *i*, the expectation of the score *s*_*i*_ is the *i*/(2*n*+1)-quantile *q*_*i*_ of the standard normal distribution *N*(0,1) times *σ*. In other words, *s*_*i*_/*q*_*i*_ is an estimator 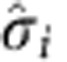 for *σ*, for each *i*. These estimators are computed only for the indices *i* in the interval (0.15*n*, 0.85*n*), that is, we consider only the middle 70% of the data to estimate *σ*. We assume that the scores *s*_*i*_ are drawn for a normal distribution only if the s.d. of the 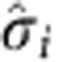 is at most 0.05 times the mean 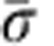 of 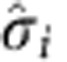; in this case, the estimators 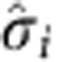 are considered to be consistent and 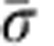 a good estimate of *σ*. If the s.d. is larger, we abandon the parametric approach and just set 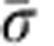 to be the s.d. of sample (*s*_1_,..., *s*_*n*_, –*s*_1_,..., –*s*_*n*_). The estimate 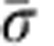 of *σ* obtained from the single-array data is used to compute the normalized *z*-score transformation 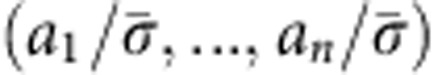 of the adjusted MADL scores (*a*_1_,..., *a*_*n*_).

### Standard assay for yeast 3β-hydroxysteroid-dehydrogenase/C4-decarboxylase

Wild-type yeast (FL200) microsomes were prepared as follows. Yeast cells were disrupted by glass bead homogenization (0.45-mm diameter) in 100 mM phosphate buffer (pH 7.5) containing 1.5 mM reduced glutathione and 30 mM nicotinamide for 8 min at 0 °C^44^. The cell extract was obtained by centrifugation at 10,000 × g for 20 min. Microsomes were then isolated by centrifuging the cell extract supernatant at 100,000 × g for 90 min. The resulting microsomal pellet was resuspended in 100 mM phosphate buffer (pH 7.5) containing 3 mM reduced glutathione and 20% glycerol (v/v) using an Elvehjem–Potter homogenizer. Yeast microsomes (1.2 mg of protein) were incubated in the presence of 4α-carboxy-4β-methyl-cholest-8,24-dien-3β-ol (50–300 mM) and NAD^+^ (0.5 mM) for 35–45 min at 30 °C. After addition of coprostanone (2–6 μg) as internal standard, the reactions were extracted as described previously^12^. The extract was analysed by TLC on silica gel and eluted twice with CH_2_Cl_2_. The fraction migrating between a standard of 4-desmethyl-sterone (*R*_F_= 0.50) and 4,4-dimethyl-sterone (*R*_F_=0.70) was eluted and analysed using a GC-MS spectrometer (Agilent 5973 N).

In the case of inhibition assays, microsomes were incubated for 35–45 min at 30 °C in the presence of 4α-carboxy-4β-methyl-cholest-8,24-dien-3β-ol (50–300 mM) and a range of inhibitor concentration (0.01–100 μM) from which a dose–response curve was obtained allowing the corresponding I_50_ values to be determined.

### GC-MS analysis for 3β-hydroxysteroid-dehydrogenase/C4-decarboxylase assay

The GC-MS spectrometer (Agilent 5973 N) was equipped with an ‘on column' injector and a capillary column (30 × 0.25 mm) coated with DB5 (H_2_ flow rate of 2 ml min^−1^). The temperature program included a 30 °C min^−1^ increase from 60 °C to 240 °C, and followed by a 2 °C/min increase from 240 °C to 280 °C. The areas of the GC peaks of coprostanone (*t*_R_=1.000) and of the produced 4α-methyl-cholest-8,24-dien-3-one (*t*_R_=1.139), corrected from endogenous component of the same *t*_R_ (1.139) determined in the blank (boiled microsomes), allowed the rate of transformation of 4α-carboxy-4β-methyl-cholest-8,24-dien-3β-ol to be measured. Under these conditions, the estimated limit of detection of the 3βHSD/D activity was 0.1 nmol h^−1^ mg^−1^.

### Molecular modelling

A homology model was produced by using *P. aeruginosa UDP-N*-acetylglucosamine 4-epimerase complexed with UDP-N-acetylgalactosamine and NAD+ (1SB8) as a template, due to its highest primary sequence similarity (24%) compared with all the other structures in PDB. The sequence alignment was performed by ClustalX^45^ and the homology model was constructed by MODELLER^46^. The model was then checked by QMEAN^47^ (http://swissmodel.expasy.org/qmean/cgi/index.cgi).The structural comparison of the homology model with the other structures in the PDB was performed by DaliLite^48^ (http://www.ebi.ac.uk/Tools/structure/dalilite/). All the macromolecular figures were generated in PyMol (version 1.3; Schrödinger, LLC).

The QMEAN score and the *Z*-score for the model are 0.475 and −3.46 respectively. QMEAN score is global score for the whole model and reflects the predicted model reliability ranging from 0 to 127. The QMEAN *Z*-score estimates the absolute quality of a homology model by relating it to reference structures solved by X-ray crystallography. These reference structures are a non-redundant subset of the PDB sharing less than 30% pairwise sequence identity among each other and are solved at a resolution below than 2 Å. It is an estimate of the ‘degree of nativeness' of the structural features observed in a model. The scores are not ideal because of the absence of any other closely related protein structures to compare with the structure used to build the model with *P. aeruginosa* UDP-N-acetylglucosamine 4-epimerase.

The homology model shows conserved structural domains with other templates in Protein Data Bank such as UDP-galactose 4-epimerase mutant (PDB ID: 3AW9), Thermus thermophilus HB8 UDP-glucose 4-epimerase complex with NAD (PDB ID: 2P5U), DesIV from Streptomyces venezuelae with NAD and TYD bound (PDB ID: 1R66) (Supplementary Fig.3). The structural comparision of the homology model with the above-mentioned templates using DaliLit program generated Z-score. The Z-scores for these are 34.9 with 2.2A° RMSD, 36.9 with 1.7A° RMSD and 37.1 with 1.8A° RMSD respectively.

## Additional information

**Accession codes.** Crystallographic data (excluding structure factors) have been deposited with the Cambridge Crystallographic Data Centre as supplementary publication number CCDC 976241. These data can be obtained free of charge from the Cambridge Crystallographic Data Centre via www.ccdc.cam.ac.uk/data_request/cif

**How to cite this article:** Helliwell, S. B. *et al*. FR171456 is a specific inhibitor of mammalian NSDHL and yeast Erg26p. *Nat. Commun.* 6:8613 doi: 10.1038/ncomms9613 (2015).

## Supplementary Material

Supplementary Information Supplementary Figures 1-10, Supplementary Table 1-4 and Supplementary References.

Supplementary Dataset 1 Metabolomics dataset

## Figures and Tables

**Table 1:** Crystal data and structure refinement for FR171456.

Empirical formula	C_31_ H_48_ O_8_
Formula weight	548.69
Temperature	100(2) K
Wavelength	1.54178 Å
Crystal system	Monoclinic
Space group	P21
Unit cell dimensions	*a*=7.447(2) Å
	*b*=10.193(2) Å
	*c*=19.229(3) Å
Volume	1,439.1(5) Å^3^
*Z*	2
Density (calculated)	1.266 g cm^−^^3^
Absorption coefficient	0.730 mm^−1^
F(000)	596
Crystal size	0.30 × 0.18 × 0.09 mm^3^
Theta range for data collection	2.33–68.20°
Index ranges	−8≤*h*≤8, −11≤*k*≤10, −23≤l≤23
Reflections collected	44,812
Independent reflections	5,008 [R(int)=0.0347]
Completeness to theta=68.20°	99.3%
Absorption correction	Semi-empirical from equivalents
Max. and min. transmission	0.9372 and 0.8108
Refinement method	Full-matrix least-squares on F^2^
Data/restraints/parameters	5,008/13/399
Goodness-of-fit on F^2^	1.048
Final R indices (*I*>2sigma(*I*))	R1=0.0276, wR2=0.0704
R indices (all data)	R1=0.0279, wR2=0.0708
Absolute structure parameter	0.06(10)
Largest difference peak and hole	0.168 and −0.177 e.Å^−3^

max., maximum; min., minimum.
