# Supplementary material for: FR171456 is a specific inhibitor of mammalian NSDHL and yeast Erg26p
Source: Nat Commun. 2015 Oct 12;6:8613. doi: 10.1038/ncomms9613 (PMC4633953; doi:10.1038/ncomms9613)
Supplement: Supplementary Information — Supplementary Figures 1-10, Supplementary Table 1-4 and Supplementary References. [file ncomms9613-s1.pdf]

Supplementary figure 1

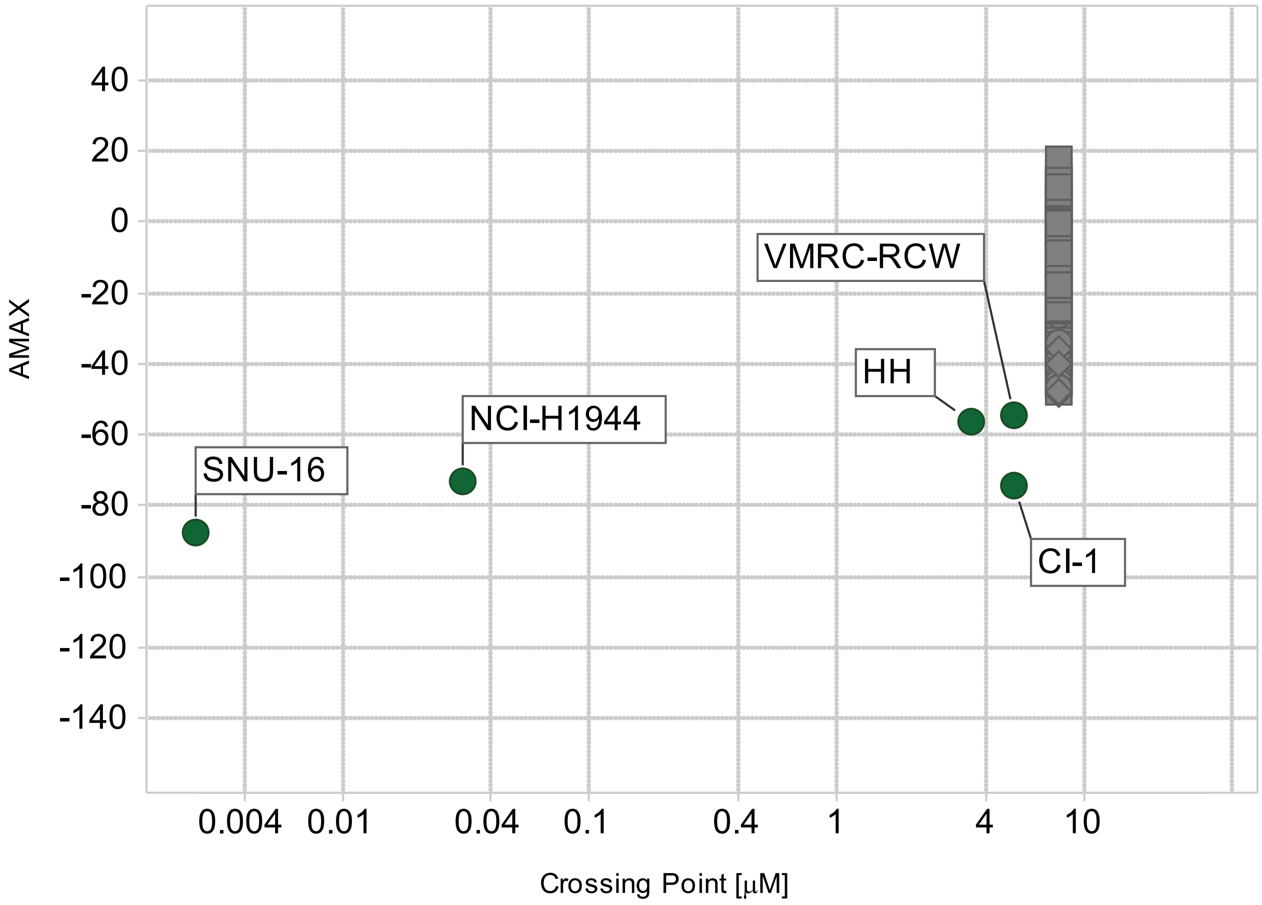

Supplementary figure 1. FR171456 is not anti-proliferative in mammalian cells in culture. A plot of normalized maximal inhibition (Y axis) versus the inflection point (X Axis) of a standard dose-response curve for increasing concentrations of FR171456 incubated with 503 mammalian cell lines in culture for 72h (each symbol), as determined by Cell TiterGlo (Promega) readout.

Supplementary figure 2

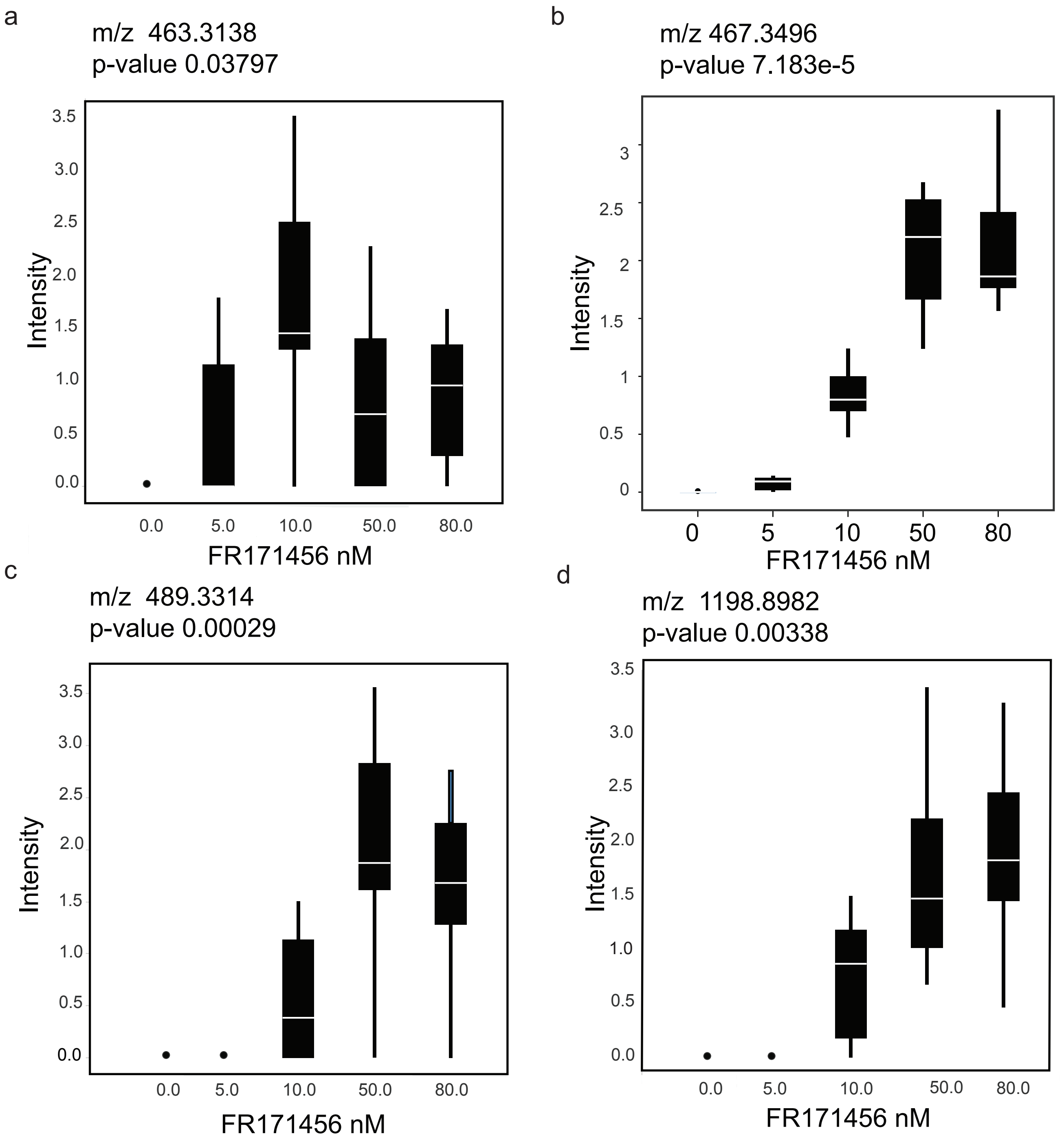

Supplementary figure 2. Additional metabolites increase following FR171456 treatment. Plot of peak intensities (Y axis) versus concentration of FR171456 (nM)(X Axis) for metabolites with m/z of (a) 463.3183, (b) 467.3496(c) 489.3314 and (d) 1198.8982. Bars and sticks represent 25/75th percentile and 1.58 times the interquartile range, respectively, from 15 separate measurements.

Supplementary figure 3

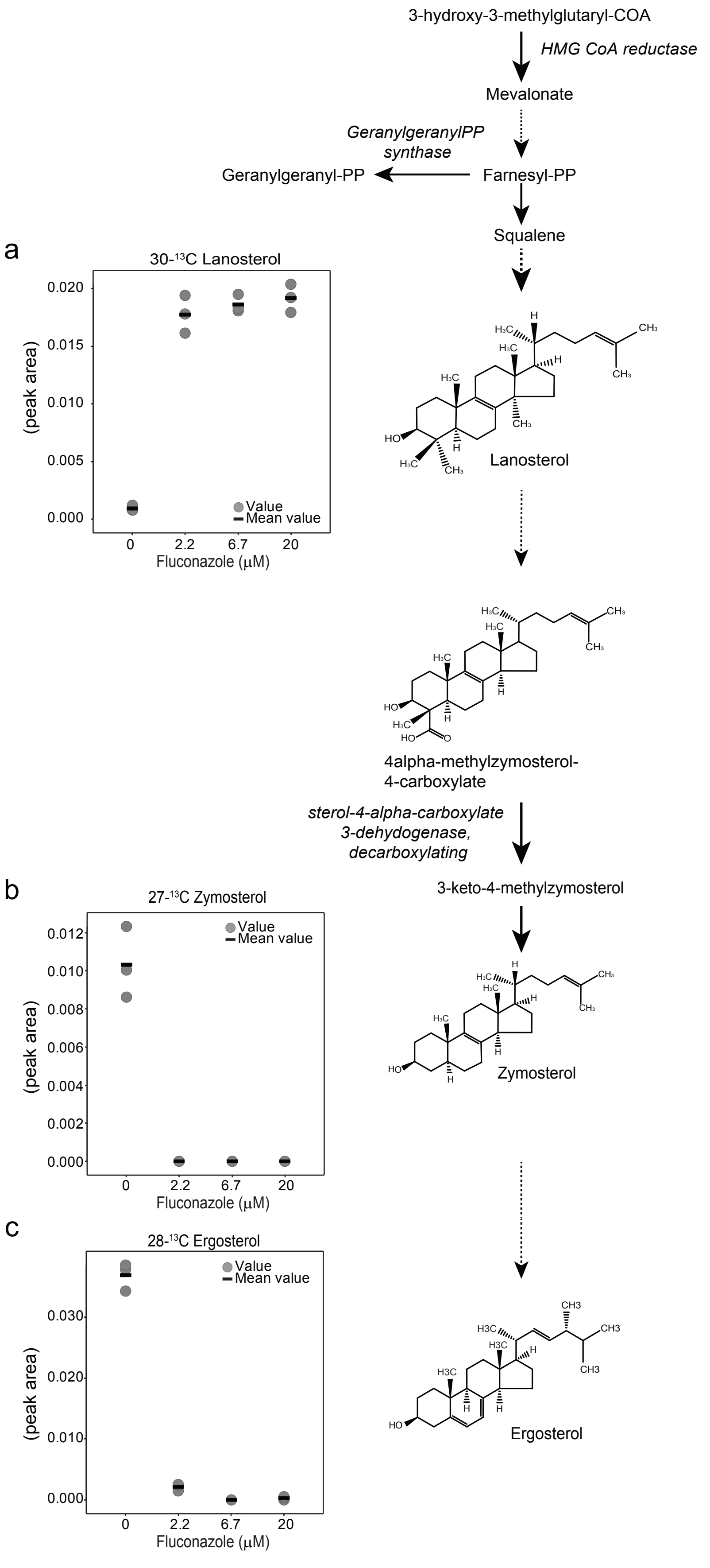

Supplementary figure 3. Fluconazole treatment inhibits Erg11p of *C. albicans*. The sterol pathway is shown highlighting selected products / substrates of pathway enzymes that could be detected as significantly altered in *C. albicans* cells treated with increasing doses of fluconazole for 14 h (a, b, and c) shown are values (grey circles) and mean of values (black bar) for three biological replicates.

Supplementary figure 4

a

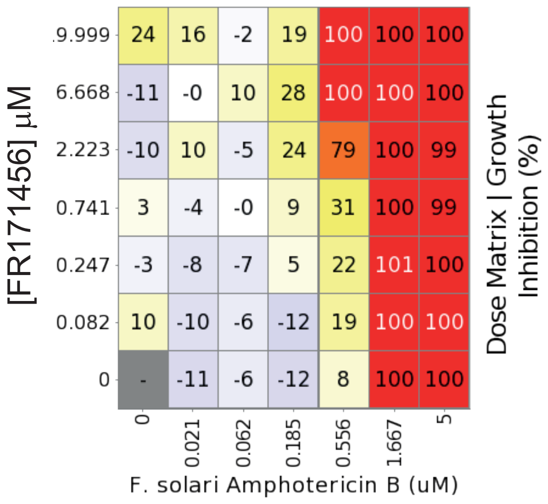

b

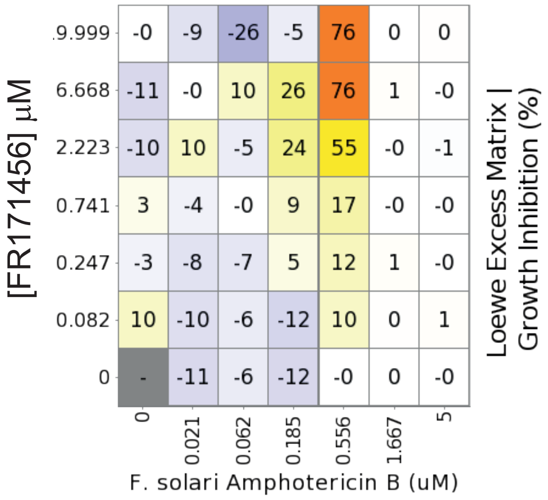

c

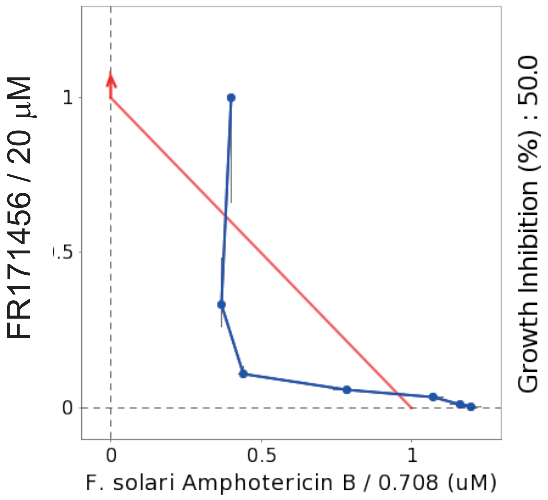

Supplementary figure 4. FR171456 is synergistic with Amphotericin B on *F. solani*. *F. solani* cells were incubated for 24 h in increasing combined doses of FR171456 and Amphotericin B, and growth assessed by OD530. (a) Normalised growth rates plotted as % inhibition of growth. (b) Loewe excess heat map showing difference between predicted scores based upon the addition of each agent alone and the actual values detected plotted as difference in % of inhibition. (c) Loewe isobologram - a comparison of the doses of each compound needed to achieve 50% inhibition of proliferation.

Supplementary figure 5

```
S.cerevisiae      ----------------------------------MSKIDSVLIIGGSGFLGLHLIQQFFD
C.albicans        ---------------------------------MSESLQSVLIIGGSGFLGLHSIEQFYR
A.fumigatus       ----------------------------MPQKRPTLELGSVLVVGGCGFLGWHIVDQLLN
A.thaliana        -----------------------------MVMEVTETERWCVVTGGRGFAARHLVEMLVR
Mouse             MEQAVHGESKRGQVTGTHL------------TNNISKAKKCTVIGGSGFLGQHMVEQLLE
Human             MEPAV-SEPMRDQVARTHLTEDTPKVNADIEKVNQNQAKRCTVIGGSGFLGQHMVEQLLA
                                                          : ** ** . * :: :

S.cerevisiae      INPKPDIHIFDVRD--------------LPE-------KLSKQFTFNVDDIKFHKGDLTS
C.albicans        HCPNVAITVFDVRP--------------LPE-------KLSKYFTFDPSKIQFFKGDLTS
A.fumigatus       FPSETDASVALPKPEGDSRFDNPRLADRYPRCVAKVSVLDLRTANNRLPGAQYYDGDITS
A.thaliana        YQM-FHVRIADLAPAIV--------LNPHEE-------TGILGEAIRSGRVQYVSADLRN
Mouse             RGY--TVNVFD------------------------------IHQGFDNPRVQFFIGDLCN
Human             RGY--AVNVFD------------------------------IQQGFDNPQVRFFLGDLCS
                          :                                          ::  .*: .

S.cerevisiae      PDDMENAINESKANVVVHCASPMHG-QNPDIYDIVNVKGTRNVIDMCKK------CGVNI
C.albicans        DKDVSDAINQSKCDVIVHSASPMHG-LPQEIYEKVNVQGTKNLLSVAQK------LHVKA
A.fumigatus       EESLLAIFRKVKPDVVIHTATANVLEGNKELLRKVNVDGTKTLLEVAGGDRGDWGGKCKA
A.thaliana        KTQVVKGFQ--GAEVVFHMAAPDSSINNHQLQYSVNVQGTTNVIDACIE------VGVKR
Mouse             QQDLYPALK--GVSTVFHCASPPPYSNNKELFYRVNFIGTKTVIETCRE------AGVQK
Human             RQDLYPALK--GVNTVFHCASPPPSSNNKELFYRVNYIGTKNVIETCKE------AGVQK
                    .:   :.    ..:.* *:        ::   **  ** .::. .           :

S.cerevisiae      LVYTSSAGVIFNGQDV-HNADETWPIPE--VPMDAYNETKAIAEDMVLKANDPS-SDFYT
C.albicans        LVYTSSAGVIFNGQDV-INADETWPYPE--VHMDGYNETKAAAEEAVMKANDN--DQLRT
A.fumigatus       FVYTSSASVLHDTQSDLKNVNEDWPLIRGKLQLEYYSDTKAEAEEIVLKYNRASPSSMVT
A.thaliana        LIYTSSPSVVFDGVHGTLNADESLPYPP--KHNDSYSATKAEGEALILKAN-GR-SGLLT
Mouse             LILTSSASVVFEGVDI-KNGTEDLPYAM--KPIDYYTETKILQERAVLDANDPK-KNFLT
Human             LILTSSASVIFEGVDI-KNGTEDLPYAM--KPIDYYTETKILQERAVLGANDPE-KNFLT
                  :: *** .*:.:      *  *  *        : *. **   *  ::  *    . : *

S.cerevisiae      VALRPAGIFGPGDRQLVPGLRQVA---KLGQSKFQIGDNNNLFDWTYAGNVADAHVLAAQ
C.albicans        VCLRPAGIFGPGDRQLVPGLRASA---KLGQSKYQLGDNNNLFDWTYVGNVADAHVLAAQ
A.fumigatus       CALRPAGIYGEKDTTFTFKVLEHAAKASPTVLRMQLGDNNNLFDFTYVGNVAYSHLLAAY
A.thaliana        CCIRPSSIFGPGDKLMVPSLVTAA---RAGKSKFIIGDGSNFYDFTYVENVVHAHVCAER
Mouse             AAIRPHGIFGPRDPQLVPILIDAA---RKGKMKFMIGNGENLVDFTFVENVVHGHILAAE
Human             TAIRPHGIFGPRDPQLVPILIEAA---RNGKMKFVIGNGKNLVDFTFVENVVHGHILAAE
                   .:** .*:*  *  :.  :   *        :  :*: .*: *:*:. **. .*: *

S.cerevisiae      KLLDPK-----------TRTAVSGETFFITNDTPTYFWALARTVWKADGHIDK--HVIVL
C.albicans        KILDKS-----------TRDDISGQTFFITNDSPTYFWTLARTVWKNDGYIDK--YYIKL
A.fumigatus       RLLATQTRYESGQSGPLDHEKVDGEAFNITNDSPVYFWDITRAAWALAGKVVEPNQVWQL
A.thaliana        ALASGG----------EVCAKAAGQAYFITNMEPIKFWEFMSQLLEGLGYERP---SIKI
Mouse             HLSQDA--------------ALGGKAFHITNDEPIPFWTFLSRILTGLNYEAP---KYHI
Human             QLSRDS--------------TLGGKAFHITNDEPIPFWTFLSRILTGLNYEAP---KYHI
                   :                     *::: ***  *  ** :                   :

S.cerevisiae      KRPVAICAGYLSEWVS---K-MLGKEPGLTPFRVKIVCAYRYHNIAKAKKLLGYTPRVGI
C.albicans        PYPVALTLGYISEFVA---KNILKKEPGITPFRVKVVCAIRYHNIAKAKKLLGYKPEVDL
A.fumigatus       SEDLLGPVGAVLETVF----GLIGKTPRLTRRIVRYSCMTRYYSCEKAKYRLGYSPIVSV
A.thaliana        PASLMMPIAYLVELAYKLLGPYGMKVPVLTPSRVRLLSCNRTFDSSKAKDRLGYSPVVPL
Mouse             PYWMAYYLAFLLSLLVMVVSPLIQIQPTFTPIRVALAGTFHYYSCEKAKKLFGYRPLVTM
Human             PYWVAYYLALLLSLLVMVISPVIQLQPTFTPMRVALAGTFHYYSCERAKKAMGYQPLVTM
                     :    . : .             * :*   *      : ..  :**  :** * * :

S.cerevisiae      EEGINKTLAWMDEGL---------------------------------------------
C.albicans        ETGINYTLDWMNEDL---------------------------------------------
A.fumigatus       PEGLSRAVGYVLARERLESEKKGL------------------------------------
A.thaliana        QEGIKRTIDSFSHLKAQNQPKTEVTETIQWKKQTLIAIVILITLYHNFVATTGSSSVIIT
Mouse             DEAVERTVQSFHHLRKDK------------------------------------------
Human             DDAMERTVQSFRHLRRVK------------------------------------------
                    .:. ::  .

S.cerevisiae      --------------------------------
C.albicans        --------------------------------
A.fumigatus       --------------------------------
A.thaliana        AVSKVLLVSSIFMFINGILPEKMKVFGSKKID
Mouse             --------------------------------
Human             --------------------------------
```

Supplementary figure 5. Distribution of FR171456-resistant mutations along Erg26p and homologues. Primary amino acid sequence of sterol-4-α-carboxylate 3-dehydrogenase from 6 organisms with identity (*) and similarity (:) highlighted. Bold amino acids denote those that were altered in *S. cerevisiae* Erg26p to confer resistance to FR171456 but were sufficient to allow normal growth when existing as the sole copy of Erg26p.

Supplementary figure 6

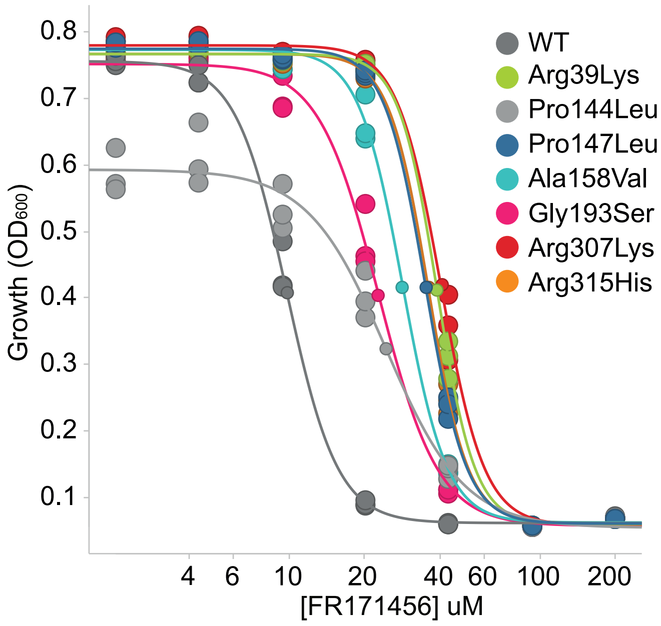

Supplementary figure 6. Multiple mutations in Erg26p confer FR171456 resistance. Dose-response curve for yeast haploid strains with single copy of ERG26 carrying single point mutations conferring resistance to FR171456 (see also Fig. 3a). Y- axis: OD600 absorbance indicating growth, X-axis: compound concentrations.

Supplementary figure 7

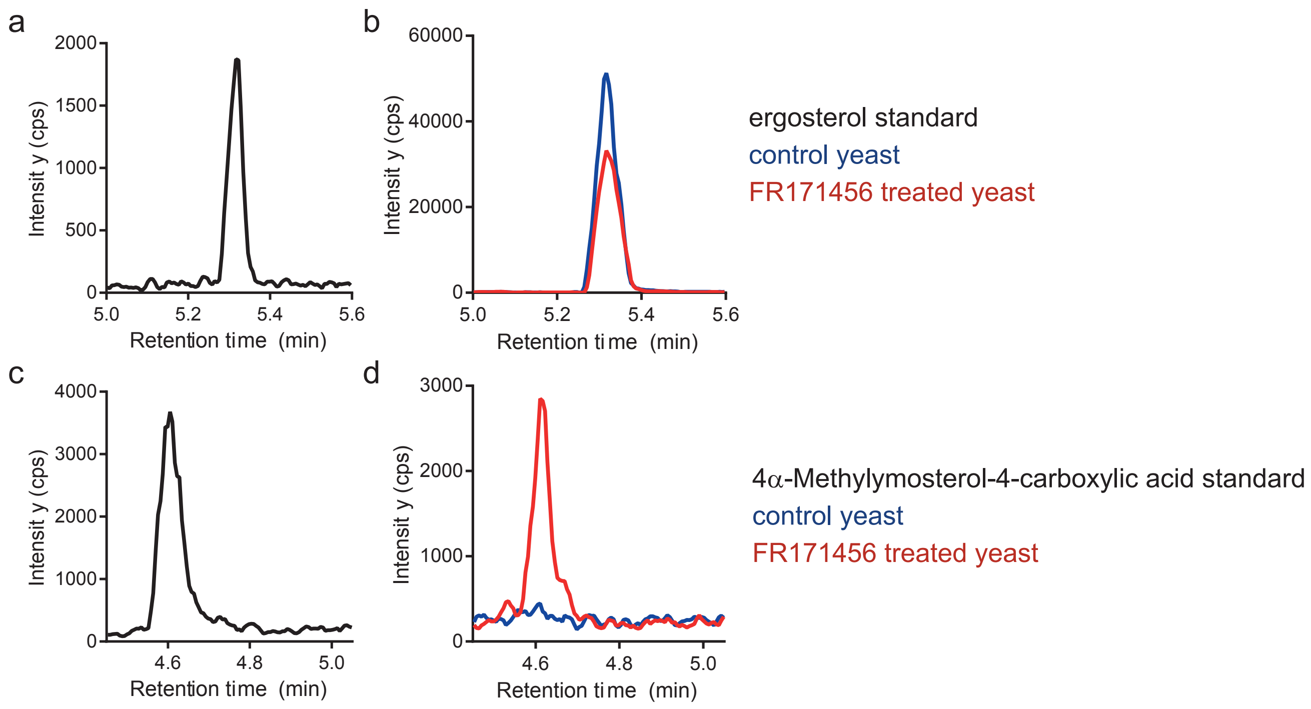

Supplementary figure 7. The Erg26p precursor accumulates upon treatment with FR171456. Metabolic profiling of *S. cerevisiae* following treatment with FR171456. (a) Chromatogram of a pure ergosterol standard monitored with the mass transition 379.3 → 69.2. (b) Chromatograms of ergosterol in lipid extracts from control yeast cells and yeast cells treated with FR171456 (1466 nM) for 7 h. (c) Chromatogram of the pure 4α-methylzymosterol-4-carboxylic acid standard monitored with the mass transition 425.3 → 109.1. (d) Chromatograms of 4α-methylzymosterol-4-carboxylic acid (MZCA) in lipid extracts from control yeast cells and yeast cells treated with FR171456 (1466 nM) for 7 h.

Supplementary figure 8

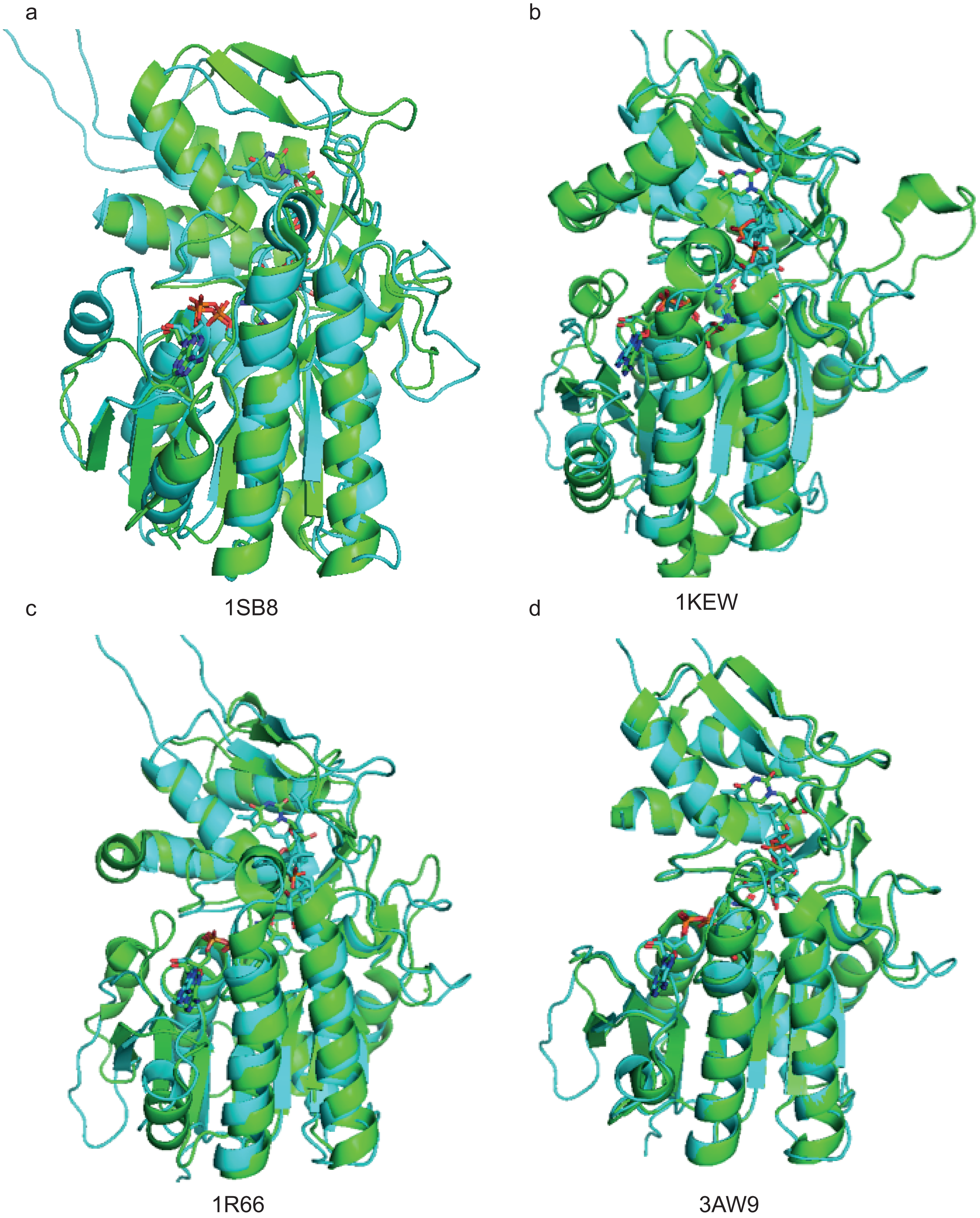

Supplementary figure 8. Homology models for Erg26 based upon 4 crystal structures. Models are shown for Erg26 lined up with (a) *Pseudomonas aeruginosa* UDP-N-acetylglucosamine 4-epimerase complexed with UDP-N-acetylgalactosamine (1SB8), (b) dTDP-D-glucose 4,6-dehydratase (RmlB) from *Salmonella enterica serovar Typhimurium* with thymidine diphosphate bound, (c) DesIV from *Streptomyces venezuelae* with NAD and TYD bound (PDB ID: 1R66) and (d) UDP-galactose 4-epimerase mutant from *Pyrobaculum calidifontis* (PDB ID: 3AW9).

Supplementary figure 9

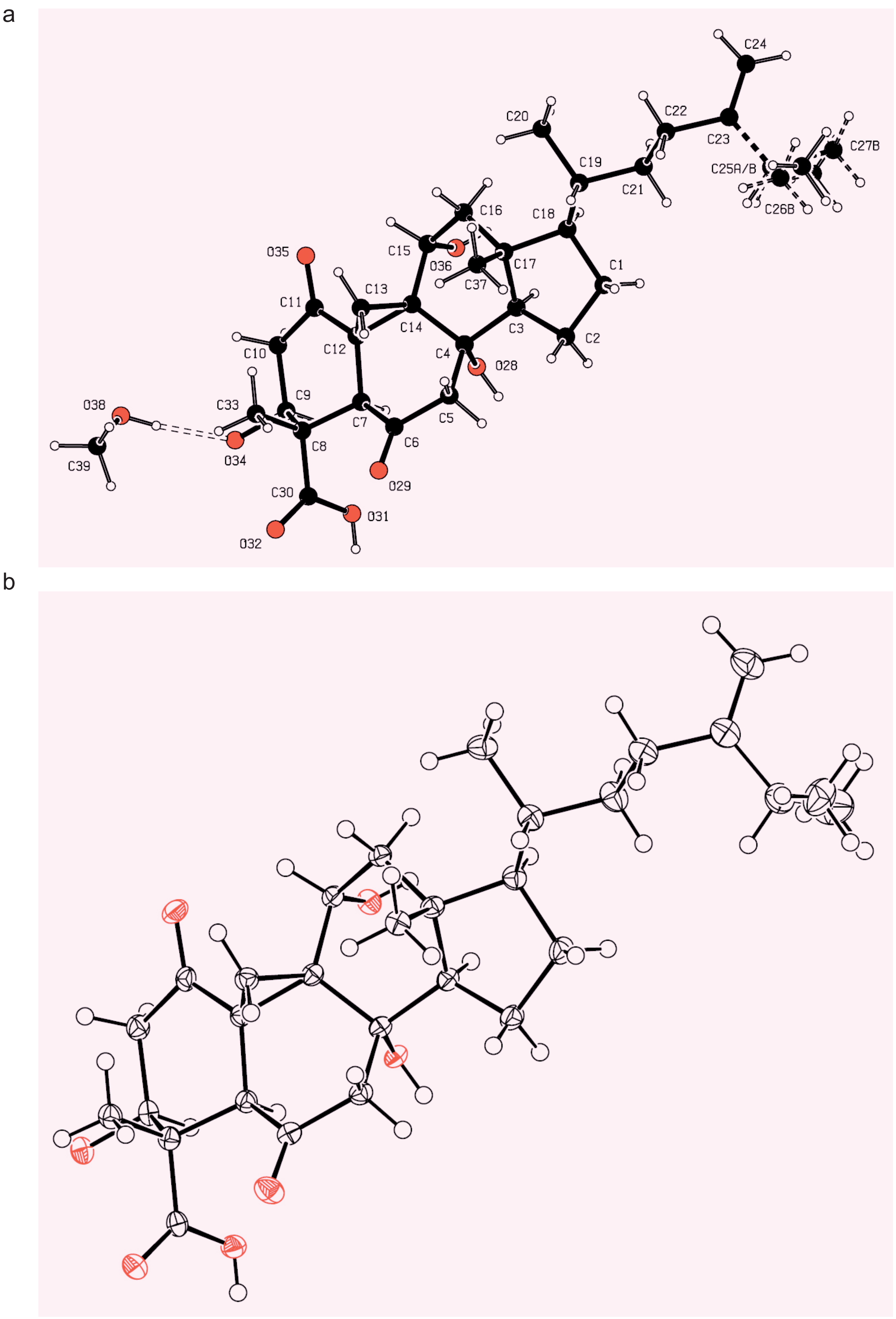

Supplementary figure 9. Two representations of the X-ray structure of FR171456.
(a) Backbone number assignments and (b) ORTEP plot.

Supplementary figure 10.

a

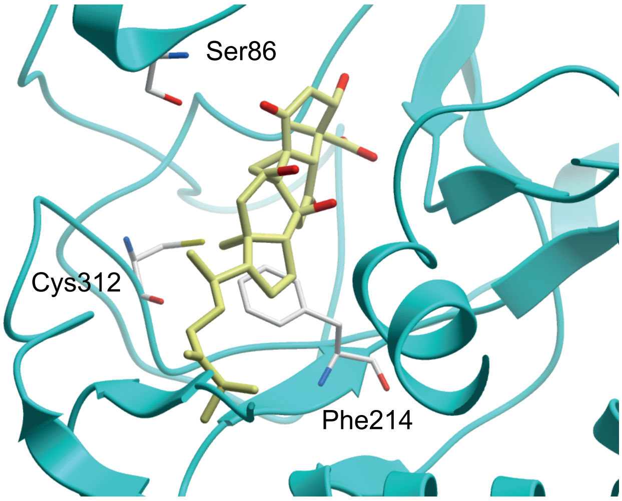

Supplementary figure 10. Some FR171456-resistant mutations likely alter NAD+ interactions with Erg26p. Homology modeling of *S. cerevisiae* Erg26p with a close up of the NAD+ binding site showing the interactions of NAD+ with Ser86, Phe214 and Cys312.

Supplementary tables:

Supplementary Table 1. The significant metabolites identified by metabolomics profiling of Huh-7 human cells exposed to FR171456.

m/z	Fold change	p-value	Predicted formula	ppm	Possible identification	KEGG ID
401.3418	0.680	0.2009	$C_{27}H_{44}O_2$ (+H^+)	-0.93	calcidiol, 7α-hydroxycholest-4-en-3-one	C05455, C01561
463.3183	infinite	0.03797	$C_{29}H_{44}O_3$ (+Na^+)	-0.086	pfaffic acid	C08965
465.3339	822.2	0.00209	$C_{29}H_{46}O_3$ (+Na^+)	0.068	4α-methylzymosterol-4-carboxylate	C15808
467.3496	3675.4	0.00047	$C_{29}H_{48}O_3$ (+Na^+)	-0.092	3β-hydroxy-4β-methyl-5α-cholest-7-ene-4α-carboxylate	C04840
489.3314	infinite	0.00029	$C_{28}H_{44}N_2O_5$ (+H^+)	1.91	-	-
1198.8982	9665.6	0.00338	-		-	-

Supplementary Table 2. FR171456 anti-fungal activity reported as MIC in µg/ml and the concentration in µM is also included[2,3].

Species	FR171456
Saccharomyces cerevisiae NF3201	8 (18 µM)
Candida albicans NF2103	32 (72 µM)
Aspergillus fumigatus NF4905	16 (36 µM)
Rhizopus oryzae NF4801	8 (18 µM)
Candida tropicalis NF2903	32 (72 µM)
Candida krusei NF2902	64 (144 µM)
Candida parapsilosis NF2903	32 (72 µM)
Candida glabrata NF2905	16 (36 µM)
Cryptococcus neoformans NF3102	32 (72 µM)
Aspergillus favus NF4902	64 (144 µM)
Aspergillus terreus NF4906	4 (9 µM)
Fusarium verticillioides NF4901	32 (72 µM)
Scedosporium apiospermum NF4908	64 (144 µM)

Supplementary Table 3. List of FR171456 resistance mutants in yeast *ERG26* and their corresponding frequency following random mutagenesis.

Codon alteration of isolated allele in a random mutagenesis experiment	Number of times isolated
Wild type	7
Arg39Lys	2
Gly90Ser	2
Pro144Leu	3
Pro147Leu	4
Ala158Val	1
Gly193Ser	1
Arg307Lys	10
Arg315His	10

Supplementary Table 4: Multiple reactions monitoring transition on the API 4000 Triple Quadrupole mass spectrometer.

Compound	Q1	Q3	DP	CE	CXP
Ergosterol	379.5	69.1	118	45	12.2
28 C^{13}-ergosterol	407.5	74.1	118	45	12.2
Lanosterol	409.5	69.1	106	52	12.2
30 C^{13}-lanosterol	439.5	74.1	106	52	12.2
Squalene	411.4	231.3	75	25	19
30 C^{13}-squalene	441.1	248.3	75	25	19
Zymosterol	367.5	95.2	112	48	5.1
27 C^{13}-zymosterol	394.5	102.1	112	48	5.1

Supplementary references

1 Barretina, J. *et al.* The Cancer Cell Line Encyclopedia enables predictive modelling of anticancer drug sensitivity. *Nature* **483**, 603-607 (2012).
2 CLSI. Reference method for broth dilution antifungal susceptibility testing of filamentous fungi; approved standard, 2nd ed. CLSI document M38-2A. (2008).
3 CLSI. Reference method for broth dilution antifungal susceptibility testing of yeasts; approved standard, 3rd ed. CLSI document M27-A3. (2008).
